# Axially Growing Carbon Quantum Ribbon with 2D Stacking Control for High‐Stability Solar Cell

**DOI:** 10.1002/advs.202400817

**Published:** 2024-07-19

**Authors:** Yuxin Shi, Yongshuai Gong, Yang Zhang, Yunchao Li, Xiaohong Li, Zhan'ao Tan, Louzhen Fan

**Affiliations:** ^1^ Key Laboratory of Theoretical & Computational Photochemistry of Ministry of Education College of Chemistry Beijing Normal University Beijing 100875 China; ^2^ Beijing Advanced Innovation Center for Soft Matter Science and Engineering Department Beijing University of Chemical Technology Institution Beijing 100029 China

**Keywords:** 2D film, axially growing, carbon quantum ribbon, solar cell, stacking model

## Abstract

Although power conversion efficiency (PCE) of solar cells (SCs) continues to improve, they are still far from practical application because of their complex synthesis process, high cost and inferior operational stability. Carbon quantum dots with high material stability and remarkable photoluminescence are successfully used in light‐emitting diodes. A good light emitter should also be an efficient SC according to the photon balance in Shockley–Quieisser formulation, in which all excitons are ultimately separated. However, the finite quantum‐sized *sp* ^2^ domain leads to tight exciton bonding, and highly delocalized electron clouds in irregular molecular stacks form disordered charge transfer, resulting in severe energy loss. Herein, an axially growing carbon quantum ribbon (AG‐CQR) with a wide optical absorption range of 440–850 nm is reported. Structural and computational studies reveal that AG‐CQRs (aspect ratio ≈2:1) with carbonyl groups at both ends regulate energy level and efficiently separate excitons. The stacking‐controlled two‐dimensional AG‐CQR film further directionally transfers electrons and holes, particularly in AB stacking mode. Using this film as active layer alone, the SCs yield a maximum PCE of 1.22%, impressive long‐term operational stability of 380 h, and repeatability. This study opens the door for the development of new‐generation carbon‐nanomaterial‐based SCs for practical applications.

## Graphical abstract

Axially growing carbon quantum ribbons (AG‐CQRs) are obtained with carbonyl groups and stacking‐controlled two‐dimensional  AG‐CQR film that achieved efficient charge separation and directional exciton transport. A solar cell based on this film as the active layer alone yielded a maximum power conversion efficiency of 1.22%, impressive long‐term operational stability of 380 h, and repeatability.

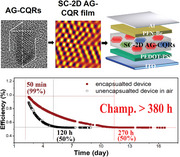

## 1 Introduction

Conversion of sunlight into electrical power has attracted considerable interest. In the 1950s, first solar cells (SCs) based on organic small molecules showed a power conversion efficiency (PCE) of less than 0.1%. These SCs have now achieved breakthrough PCEs exceeding 20%, an accomplishment attained over the past decades.^[^ ^1^, ^2^, ^3^, ^4^ ^]^ However, maintaining their performance during long‐term operation is still challenging for the reported materials, such as perovskite crystals or organic molecules, which seriously hinders further practical applications in commercialization.^[^ ^5^, ^6^, ^7^, ^8^ ^]^ Therefore, high‐quality materials should be urgently developed to overcome this limitation. Owing to high photo/thermal stability, tunable luminescence, excellent solution processability, low cost, and low toxicity, carbon quantum dots (CQDs) have been successfully used in optoelectronics, such as light‐emitting diodes.^[^ ^9^, ^10^, ^11^, ^12^, ^13^ ^]^ A good light emitter should also be an efficient SC according to the detailed balance in the Shockley–Quieisser formulation, in which an efficient external luminescence is a necessity for achieving low internal optical losses.^[^ ^14^, ^15^, ^16^, ^17^, ^18^ ^]^ For instance, carbon dot (CD)‐supported silver nanoparticles can serve as an interfacial layer to improve the device performance of polymer SCs. The photoluminescence (PL) intensity of films with such nanoparticles is ≈30% higher than that of films without the nanoparticles.^[^ ^16^ ^]^ Moreover, CDs can also be incorporated into the device architecture of perovskite SCs to improve long‐term stability and reduce toxicity, further indicating the significant development potential of CDs in SCs.^[^ ^3^, ^19^ ^]^

However, considering that all photogenerated excitons should ultimately be separated, dissociated into free charge carriers (holes and electrons) by diffusion, and extracted by the collecting electrodes, CQDs as the active layer in SCs face a series of issues. Due to the quantum confinement effect, quantum‐sized CQDs demonstrate discrete energy levels, leading to narrow bandwidth and extremely low energy absorption efficiency across the entire visible spectrum (≈400–800 nm).^[^ ^20^ ^]^ Moreover, considering the exact structure of CQDs in a finite *sp* ^2^ conjugate plane, excitons are inclined to be more strongly localized in a single dot, resulting in tightly bonded excitons that are hard to separate into electrons and holes.^[^ ^11^ ^]^ Furthermore, CQDs generally have an irregular atomic arrangement and uncontrolled *π*–*π* aggregation, which leads to a highly delocalized electron cloud for disordered charge transfer.^[^ ^21^, ^22^ ^]^ Therefore, breaking the limitations of quantum‐sized and disordered *π*‐aggregation of CQDs is the key step to effectively separate electrons and holes and directionally transfer excitons.

Generally, in networks consisting of shorter molecules, electron transport is dominated by hopping. Whereas, band conduction is prevalent in networks consisting of longer molecules, as shown in organic SCs with D‐*π*‐A molecular design.^[^ ^23^, ^24^, ^25^ ^]^ Based on this, directional axial growth of carbon core with strong electron‐withdrawing functional groups can achieve band conduction by effectively regulating the distribution of electron cloud in CQDs. This can efficiently drive excitons to dissociate into holes and electrons in addition to significantly controlling the energy gap (∆*E*) between the highest occupied molecular orbital (HOMO) and the lowest unoccupied molecular orbital (LUMO). Moreover, with the charge diffusion to the molecular interfaces, specific structural stacking of *π*‐conjugated backbone, such as edge‐on and face‐on,^[^ ^25^ ^]^ can directionally transport charge to achieve efficient extraction.

Here, we report axially growing carbon quantum ribbons (AG‐CQRs) obtained by the judicious selection of 5,7,12,14‐pentacenetetrone (PT) with high charge mobility and specific active reaction sites on the benzene ring and carbonyl groups as precursors. AG‐CQRs (aspect ratio ≈2:1) modified with carbonyl groups at both ends have a wide bandgap absorption range of 440–850 nm and can efficiently separate electrons and holes by inducing the distribution of electron clouds. The specific molecular stacking between the AG‐CQRs forms a stacking‐controlled two‐dimensional AG‐CQR (SC‐2D‐AG‐CQR) film, in which the AB mode is found to be the most stable stacking configuration. This can induce the directional transfer of excitons, resulting in a high and matched electron (*μ*_e_) and hole mobilities (*μ*_h_) of 2.03 × 10^−3^ cm^2^ V^−1^ s^−1^ and 1.82 × 10^−3^ cm^2^ V^−1^ s^−1^, respectively. SCs based on this film displays a high device performance with a PCE of 1.22%, a long operational lifetime of up to 380 h, and high measurement repeatability, indicating the high device stability and significant development potential of carbon nanomaterials for new‐generation SCs.

## 2 Results and Discussion

### 2.1 Design and Structural Characterizations of AG‐CQRs

**Figure 1:**
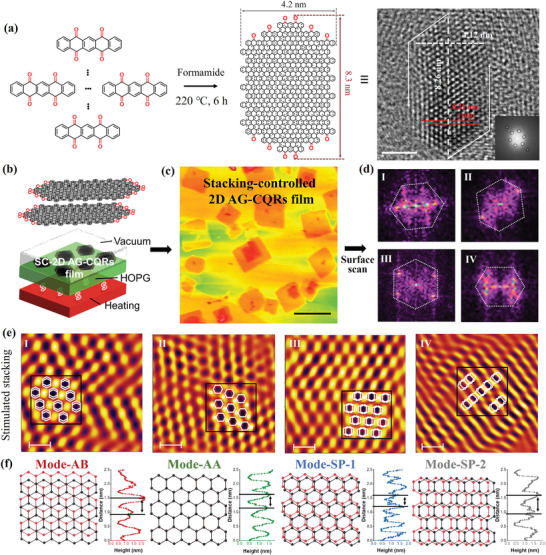
Synthesis and structural characterizations of the AG‐CQRs. a) Synthesis route, structural model, the AC‐TEM image of AG‐CQRs (Scale bar, 2 nm). b) Fabrication method and c) large‐scale AFM image of the SC‐2D AG‐CQR film (Scale bar, 1 µm). d) FFT patterns of four stacking modes and e) simulated surface topographies through the inverse FT analysis of the FFT patterns from HR‐AFM images (the solid lines represent the corresponding benzene ring configuration). Scale bar, 10 nm. f) Different stacking configurations between two AG‐CQRs, including modes AB, AA, SP‐1, and SP‐2, and corresponding height curves of the HR‐AFM images.

As shown in **Figure** **1**a, the synthesis of the AG‐CQRs involved the solvothermal treatment of PT and concentrated sulfuric acid as catalyst at 220 °C for 6 h in formamide, followed by purification via water washing, dialysis, and silica gel column chromatography (see Methods for more details). The typical spherical aberration‐corrected transmission electron microscopy (AC‐TEM) image of AG‐CQRs (Figure 1a) demonstrated a hexagonal shape with an aspect ratio of ≈2:1, in which the length and width were ≈8.26 nm and ≈4.12 nm, respectively (black lines in Figure 1a). The sixfold symmetric fast Fourier transform (FFT) patterns of the AC‐TEM image (inset in Figure 1a), and the identical well‐resolved lattice fringes with a spacing of 0.21 nm corresponding to (100) inter‐planar spacing (red lines in Figure 1a) demonstrated the nearly defect‐free crystalline structure of AG‐CQRs.

The structural characteristics of the AG‐CQRs were investigated in detail (Figure S1, Supporting Information). The Raman spectrum showed that the crystalline G band at 1615 cm^−1^ is stronger than the disordered D band at 1380 cm^−1^ with a large G to D intensity ratio (*I*_G_/*I*_D_) of ≈2.22 (Figure S1a, Supporting Information). The powder X‐ray diffraction pattern of the AG‐CQRs displayed a narrower (002) peak centered at ≈24°, which was different from the ultra‐broad (002) peak of previously reported CQDs, further indicating the high crystallinity of the AG‐CQRs (Figure S1b, Supporting Information). Fourier transform infrared spectrum indicated that the broad absorption peak in the region of 3000–3600 cm^−1^ was associated with the stretching of aromatic O─H. Strong characteristic stretching vibration bands of C═C and C═O were present at ≈1630 cm^−1^ and ≈1240 cm^−1^, respectively (Figure S1c, Supporting Information). X‐ray photoelectron spectroscopy (XPS) demonstrated that the AG‐CQRs mainly contained C (≈88%) and O (≈12%) (Figure S1d,e, Supporting Information). The deconvoluted high‐resolution XPS spectra of C1s and O1s further showed the presence of C═C (≈284.8 eV) and C═O (≈532.4 eV) (Figure S1f,g, Supporting Information). In the ^1^H‐Nuclear magnetic resonance (NMR) spectra (DMSO‐*d*6, ppm), signals in the range of 8–10 ppm corresponded to the aromatic H on the AG‐CQRs (Figure S1h, Supporting Information). ^13^C‐NMR spectra (DMSO‐*d*6, ppm) further confirmed the obvious resonance signals of carbonyl C in the range of 162–178 ppm. In addition, a large number of signals in the range of 120–145 ppm were indicative of the formation of *sp* ^2^ domains in the AG‐CQRs (Figure S1i, Supporting Information).

The aforementioned results confirmed that the AG‐CQRs possessed a highly crystalline hexagonal shape with an aspect ratio of ≈2:1 and were modified by carbonyl groups at both ends. PT possessed a unique linear structure with reactive sites at the benzene ring and carbonyl positions. Based on the different reaction activities of these two sites, each PT reacted up and down to promote the axial growth of the carbon ribbon, thus modifying the carbonyl groups at both ends of the AG‐CQRs, as shown in Figure 1a.

### 2.2 Design and Morphology of Stacking‐Controlled 2D AG‐CQR Film

Defect‐free and highly charge‐transporting active layers play an important role in achieving high‐performance SCs. 2D atomic films with highly crystalline graphene structures possess weak van der Waals interactions between the interlayers, which could introduce a unique degree of freedom for modulating the electronic properties, even though they are completely different from the molecule itself. Based on these, a high‐quality SC‐2D AG‐CQR film was formed by completely evaporating the AG‐CQDs chlorobenzene solution in a vacuum annealer at 80 °C for 15 min at the highly oriented pyrolytic graphite (HOPG) substrate (Figure 1b). Through the large‐scale scanning of this film, a series of sheets with the size of ≈300–500 nm and a thickness of ≈3 nm were observed from high‐resolution atomic force microscopy images (HR‐AFM) (Figure 1c).^[^ ^26^, ^27^ ^]^ By further scanning the surface morphology of different sheets, four surface topographies with different periodic lattice pattern were observed (Figure S2, Supporting Information), corresponding to four characteristic FFT patterns with well‐resolved peaks (Figure 1d).

Based on the inverse FT analysis, the atoms with respect to their original positions were clearly located to simulate the four different structures observed in this film. Four high‐symmetry regions were clearly visualized: rhombic, hexagonal, and parallel lattice arrays (Figure 1e). Importantly, the superlattice pattern with atomic‐level resolution within the black lines corresponded well with the periodic configuration between the AG‐CQRs and AG‐CQRs (white lines in Figure 1e). In addition, the periodic height curves of the four HR‐AFM images were observed, which displayed different periods, including ≈0.39 nm, ≈0.58 nm, ≈0.28 nm, and ≈0.63 nm, respectively (Figure 1f; Figure S3, Supporting Information). These characteristics are consistent with these of the simulated structures of the two AG‐CQRs with different stackings in Figure 1f. Therefore, the four typical stacking modes between two AG‐CQRs were clearly defined as follows: AB mode (A atoms in one layer lied directly below or above the B atoms of the other layer), AA mode (every atom had an equivalent chemical environment for other adjacent layers), and saddle‐point stacking (SP‐1 and SP‐2) configurations (the top molecule being irregularly translated on the other one) (Figure 1f). Statistics of the surface topographies of the entire film showed that compared to the other three, the type of AB occupied the main area (≈70%), indicating that AB mode was the most stable stacking configuration in this film.

### 2.3 Optical‐Electrical Properties of the SC‐2D AG‐CQR Film

**Figure 2:**
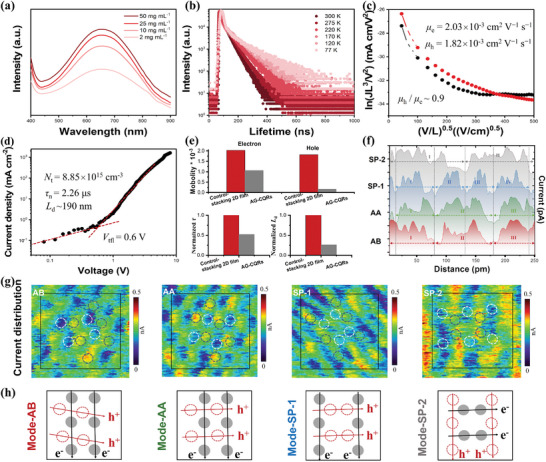
Optical‐electrical properties of the SC‐2D AG‐CQR film. a) Absorption spectra at different concentrations. b) Temperature‐dependent time‐resolved decay (77–300 K). c) *J*–*V* characteristics of electron‐only (indium tin oxide (ITO)/SnO_2_/active layer/Al) and hole‐only (ITO/poly(3,4‐ethylenedioxythiophene):poly(styrenesulfonate) (PEDOT:PSS)/active layer/MoO_3_/Al) devices. d) Dark *J*–*V* characteristics of electron‐only devices. e) Comparison of *μ*_e_ and *μ*_h_, as well as *τ*_n_ and *L*_d_ of SC‐2D AG‐CQR film and AG‐CQRs. f) The corresponding current profiles are shown according to the current images and g) high‐resolution current images (20 × 20 nm) from c‐AFM images, which show a sharp contrast in conductivity with period changes. The imaging parameters are recorded at a bias of 5 mV and a normal force of 30.5 nN. Scale bars, 5 nm. h) Schematics depicting the charge transfer paths for electrons and holes, including modes AB, AA, SP‐1, and SP‐2.

A wide absorption band from 440 nm to 850 nm was observed in the Ultraviolet‐visible (UV–vis) absorption spectrum of the SC‐2D AG‐CQR film (**Figure** **2**a). An absorption spectrum with a broad absorption range was also observed for the AG‐CQRs solution, demonstrating that the bandgap and energy levels were mainly determined by AG‐CQRs with a large‐sized and high crystalline *sp* ^2^ carbon core (Figure S4, Supporting Information). With an increase in the concentration of the AG‐CQRs solution, the intensity of the absorption bands of the films increased substantially, but the peak positions remained almost unchanged. Under different temperature from 77 K to 300 K, the UV–vis absorption spectrum had a small change in peak intensity. Notably, the peak position was well confined, which were nearly independent of the temperature (Figure S5, Supporting Information), further showing the low defect energy levels due to the nearly defect‐free structure of SC‐2D AG‐CQR film.^[^ ^12^ ^]^ From UV photoelectron spectroscopy spectra of the SC‐2D AG‐CQR film (Figure S6, Supporting Information), the energy levels (*E*) of HOMO and LUMO  were determined to be 4.02 eV and 5.39 eV, respectively. Bandgap energy of the AG‐CQRs was further calculated using the equation *E*_g_^opt^ = 1240/*λ*_edge_, where *λ*_edge_ is the onset value of the first excitonic absorption peaks in the direction of longer wavelengths. The calculated Δ*E*_g_ was 1.37 eV, which was consistent with the narrow energy gap between HOMO and LUMO.

Temperature‐dependent time‐resolved decay spectra were used to further investigate the lifetime decay of the SC‐2D AG‐CQR film (Figure 2b). Under different conditions, a longest lifetime of ≈1.8 µs was observed, which is longer than that of the reported CQDs with fluorescence emission and the neat film of those organic small molecules.^[^ ^10^, ^11^, ^12^ ^]^ The longer lifetime of orbital transition demonstrated a slow recombination rate of excitons, in which a long exciton lifetime enabled efficient exciton splitting to generate free charges while suppressing voltage losses. Moreover, the lifetime was strongly temperature‐dependent. The lifetimes decreased with increasing temperature because of a substantial increase in the non‐radiative relaxation rate, and at low temperatures, a longer lifetime indicated that the non‐radiative process was substantially suppressed.^[^ ^28^ ^]^

To explore the electrical properties of the SC‐2D AG‐CQR film, *µ*_h_ and *µ*_e_ were measured (Figure 2c). The *µ*_e_ and *µ*_h_ were found to be up to 2.03 × 10^−3^ cm^2^ V^−1^ s^−1^ and 1.82 × 10^−3^ cm^2^ V^−1^ s^−1^, with a more balanced *µ*_h_/*µ*_e_ ratio (≈0.9). The *µ*_h_ was two to three orders of magnitude higher than those of reported CQDs,^[^ ^9^, ^11^, ^12^ ^]^ which was comparable to those of reported organic transport materials (Table S1, Supporting Information). Controlled experiments were further conducted to verify that the high *µ*_h_ and *µ*_e_ were from the ordered stacking of AG‐CQRs. Conventional active‐layer films were typically fabricated by spin coating, in which only dispersed individual dots could be observed (Figure S7, Supporting Information). Furthermore, a low *µ*_h_ (1.59 × 10^−4^ cm^2^ V^−1^ s^−1^) and an imbalanced *µ*_h_/*µ*_e_ ratio (≈0.15) were observed, which was completely different from that of the SC‐2D AG‐CQR film. Compared with the individual AG‐CQRs that had a relatively high *µ*_e_, the *π*–*π* conjugate between AG‐CQRs and AG‐CQRs in this film could regulate the transfer of hole, inducing a balanced electron–hole pair.^[^ ^11^, ^29^ ^]^

Moreover, the space‐charge‐limited‐current method was used to evaluate the trap density in SCs devices. Figure 2d shows the dark current density‐voltage (*J*–*V*) curves of electron‐only devices. The linear region of Ohmic responses occurred at the low bias voltage between 0.2–0.4 V for different devices, and there was a significant increase of injected‐current at the intermediate region with the increase of bias voltage, which was defined as the trap filling process. Theoretically, the trap‐filling limit voltage (*V*_tfl_) is identified as the kink point between two regions and the trap density (*N*_t_) in the device can be calculated by the following formula^[^ ^30^ ^]^:

(1) $$N_{t}=\frac{2\;\varepsilon \;\varepsilon_{0}V_{\mathrm{tfl}}}{ed^{2}}$$

where e, *ε*, ε_0_, and *d* are the elementary charge, relative dielectric constant (generally the value is 3 for organic SCs), vacuum permittivity, and active layer thickness in devices, respectively. For devices with SC‐2D AG‐CQR film (*d* ≈ 150 nm) as the active layer, the *V*_tfl_ was 0.6 V, and the calculated *N*_t_ was 8.85 × 10^15^ cm^−3^, which were relatively low compared to those for the reported organic molecules. To better understand the direct effects of the interfacial density on charge transport, changes in electron lifetimes as a function of *N*_t_ were investigated. Theoretically, the electron lifetime *τ*_n_ can be given by the following formula^[^ ^31^ ^]^:

(2) $$\tau_{n}=\frac{1}{B_{n}N_{t}}$$

where *B*_n_ is the electron capture coefficient (*B*_n_ = 5.0 × 10^−17^ m^3^ s^−1^). *τ*_n_ is inversely proportional to *N*_t_, and *τ*_n_ is 2.26 µs. Exciton diffusion plays an important role in determining the efficiency of photoelectric conversion in OSCs. The probability that excitons diffuse to the SC‐2D AG‐CQR film interfaces is determined by the exciton diffusion length (*L*_d_), which is calculated as the following formula^[^ ^32^ ^]^:

(3) $$L_{d}=\sqrt{D\tau }$$

where D is the diffusion coefficient (≈2 × 10^−4^ cm^2^ s^−1^) and *τ* is proportional to the average PL lifetime in thin films. The maximum *L*_d_ is ≈190 nm (Figure 2e). The *L*_d_ is significantly higher than that of reported organic materials in SCs (Table S1, Supporting Information), further indicating the great charge transfer properties of SC‐2D AG‐CQR film.

The electrical conductivity map of the SC‐2D AG‐CQR film was obtained using conductive AFM (c‐AFM) images. The electrical conductivities of the four stacking modes were clearly different across the surface. The periodicity of current flow was compared (Figure 2f). In contrast to SP‐2 (two cycles) and SP‐1 (four cycles), AB and AA had three cycles, which indicated a proper current distribution with ordered transfer. However, AA exhibited a larger positive current area, which was not conducive to a balance between the positive and negative charges. Notably, the symmetrical distribution of the area in the AB cycles further proved the balanced separation of electrons and holes. This indicated that small variations in the stacking between AG‐CQRs had a significant impact on the electronic properties of the system.^[^ ^20^ ^]^ Moreover, the high‐resolution current maps indicated that a superlattice pattern also existed in the c‐AFM images (Figure 2g). The brighter lattice point (black dashed line) was more conductive than the lattice point indicated by the white lines in Figure 2g, where the unusual current enhancement corresponded well to the periodic arrangement between the AG‐CQRs in the HR‐AFM. Based on these results, the charge transfer paths in the four stacking structures were provided, in which the strong conductive points (blue points) represented the electrons, and the weak points (orange points) represented the holes (Figure 2h).^[^ ^27^ ^]^ It clearly demonstrated that the electrical properties of the SC‐2D AG‐CQR film are closely related to the electrical conductivity of the AB mode.

In summary, AG‐CQRs exhibited wide bandgap absorption (440–850 nm) and high *μ*_e_. For the SC‐2D AG‐CQR films, the four stacking modes showed different electrical properties. Among them, AB mode as the most stable stacking configuration in this film possessed a more balanced charge transfer, determining a large *τ*_n_ (≈2.26 µs), less *N*_t_ (≈8.85 × 10^15^ cm^−3^), long *L*_d_ (≈190 nm), and balanced charge mobility (≈0.9). These excellent optical and electrical properties demonstrate the considerable potential of SC‐2D AG‐CQR films for SCs.^[^ ^6^, ^7^, ^8^, ^30^, ^31^, ^32^ ^]^

### 2.4 Theoretical Investigation

#### 2.4.1 Energy Level of AG‐CQRs

**Figure 3:**
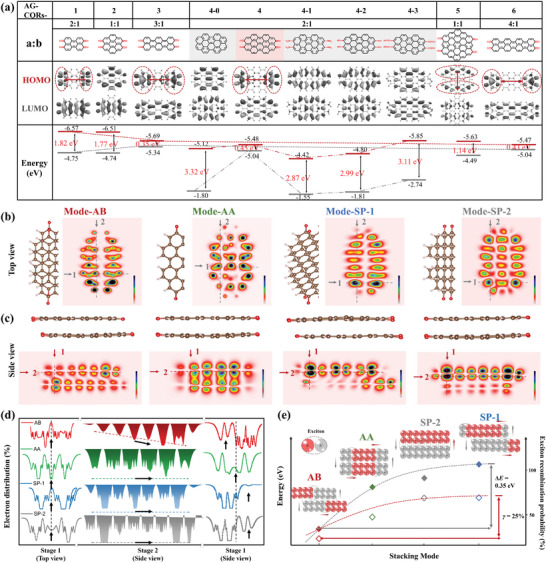
Energy level and electronic occupation calculations. Structural models of CQRs: 1) CQRs modified by carbonyl groups with different aspect ratios of 2:1, 1:1, 3:1, and 4:1, 2) CQRs with an aspect ratio of 2:1 modified by different pure CQRs, carbonyl, amino, hydroxy, and carboxyl groups. a) Schematics of the calculated HOMO, LUMO, and energy levels. Structural models of CQR‐CQR with different stacking modes, including AB, AA, SP‐1, and SP‐2, and schematics of the calculated 2D mapping of electron occupation in b) top and c) side views. d) The cycles and directions of electron distribution at different positions. e) The energy and exciton recombination probabilities in the four stacking modes; the insect is the structural diagram of the distribution and recombination of electrons and holes.

The local structure responsible for the special AG‐CQRs was further confirmed by theoretical calculations (Tables S2, Supporting Information). **Figure** **3**a shows the optimized models of AG‐CQRs modified by carbonyl groups with different aspect ratios of 2:1 (AG‐CQRs‐1 and AG‐CQRs‐4), 1:1 (AG‐CQRs‐2 and AG‐CQRs‐5), 3:1 (AG‐CQRs‐3), to 4:1 (AG‐CQRs‐6), and pure 2:1 CQRs without functional group (AG‐CQRs‐4‐0) and with different functional groups, including carbonyl (AG‐CQRs‐4), amino (AG‐CQRs‐4‐1), hydroxy (AG‐CQRs‐4‐2), and carboxyl groups (AG‐CQRs‐4‐3).^[^ ^12^, ^33^ ^]^ The *E* of HOMO and LUMO energy levels of all the structural models were calculated for comparison (Figure S8, Supporting Information). The degree of delocalization of the HOMO and LUMO were qualitatively determined, which corresponded to the electron cloud density distribution around the entire molecular structure. By increasing the proportion of axial growth from AG‐CQRs‐1, AG‐CQRs‐3, to AG‐CQRs‐6, the ∆*E* value decreased from 1.82 eV to 0.43 eV, but AG‐CQRs‐6 with fewer benzene rings at horizontal direction was not conducive to form *π*‐conjugated interaction. For the AG‐CQRs‐2/‐3 and AG‐CQRs‐5/‐6 with similar sizes but different aspect ratios, the ∆*E* for axial growth could be reduced from 1.77 eV to 0.35 eV and 1.13 eV to 0.43 eV, respectively. Moreover, the functional groups at the terminal of the AG‐CQRs, particularly the carbonyl groups, affected the distribution of the electron cloud for the change in *E*. The ∆*E* of AG‐CQRs‐4‐0, AG‐CQRs‐4‐1, AG‐CQRs‐4‐2, and AG‐CQRs‐4‐3 were very large but significantly decreased from 3.32 eV to 0.45 eV with the modification of carbonyl groups at AG‐CQRs‐4. This was induced by the large decrease of *E* of LUMO. Therefore, AG‐CQRs‐4 with carbonyl groups and an appropriate proportion of axial growth with a 2:1 aspect ratio was considered to be the most effective in regulating the *E* of the HOMO and LUMO and forming a stacking film.

#### 2.4.2 The Strategic Stacking Control Between AG‐CQRs

Subsequently, the stacking modes of the *π*–*π* conjugate in AG‐CQR and AG‐CQR were further explored to investigate the separation and transfer of electrons and holes.^[^ ^34^, ^35^, ^36^ ^]^ Figure 3b,c shows four stacking models between two CQRs, including AB, AA, SP‐1, and SP‐2 (Figure S9, Supporting Information). The electron occupation, distribution, energy, and exciton recombination were calculated for comparison (Table S3, Supporting Information). Remarkably, the electron cloud density distributions, which qualitatively represented the degree of occupation of electron or hole delocalization in the four structures, changed significantly, as shown in the 2D mapping. In the top view, the electron clouds of AB and SP‐1 are divided into two parts according to the position of the carbonyl groups and are more densely localized at one end (Figure 3b). However, for AA and SP‐2, the electron clouds were evenly distributed. In the side view, the electron clouds of the upper and lower parts of the AB were also enriched at one end in a mutually symmetrical structure (Figure 3c). However, for AA, all the two‐part electron clouds were mainly enriched at the center of the AG‐CQRs. For SP‐1 and SP‐2, the electron clouds were primarily focused on one part. Reflecting on the cycle and direction of electron distribution, AB showed obvious electron localization compared to that of other stacking structures, which was beneficial for the exciton separation of the electron–hole part (Figure 3d). Moreover, exciton separation and recombination were quantitatively demonstrated based on the distribution of the electron clouds. Theoretically, according to the distribution of the electron cloud, AB had a relatively low exciton recombination probability (≈37.5%), whereas the probability of serious recombination for the other three ranged from 50% to 67.5%. The low energy of AB (≈3.59 eV) and large energy gap (≈0.5 eV) further indicated the high stability of this stacking mode. Therefore, it can be reasonably concluded that, compared with the other three modes, the AB mode in this film was the most stable stacking structure and exhibited the strongest charge separation, as shown by the distribution of the electron cloud (Figure 3e). This is completely different from the reported CQDs with strong exciton radiative recombination and disordered exciton transport.

In conclusion, the AG‐CQRs with the carbonyl group in the direction of axial growth can lead to the electron cloud to be enriched at both ends, which plays a key role in the wide absorption and energy level matching by regulating the HOMO and LUMO to control ∆*E*. By further regulating *π*–*π* stacking between each AG‐CQRs, the electron distribution significantly induces the separation of excitons and directional transfer. The AB mode, as the most stable stacking structure, exhibits obvious charge separation and low energy, which determines the excellent electrical properties of this film, as shown by the experimental results and HR‐AFM images (Figure 1). Different with other carbon materials,^[^ ^9^, ^13^ ^]^ the unique structure and properties promote the successful fabrication of SC‐2D AG‐CQR film‐based SCs.

### 2.5 SC based on SC‐2D AG‐CQR film

#### 2.5.1 Design of Device Structure

**Figure 4:**
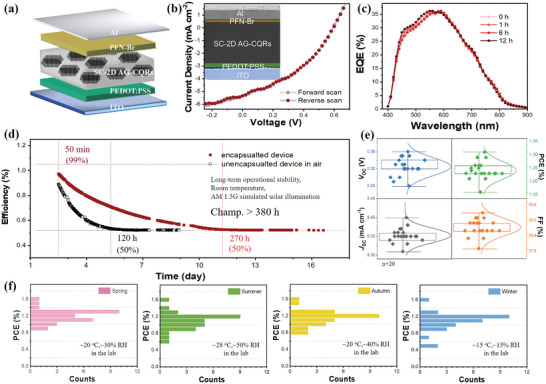
SCs structure and performance characterization. a) Device structure, b) *J*–*V* curves (inset is the corresponding cross‐sectional TEM images), and c) EQE spectra under continuous light irradiation for different times. d) Long‐term operational stability by tracking encapsulated and unencapsulated devices in the air tracking under continuous light irradiation with a white light‐emitting diode lamp. The initial efficiency of SCs is 1.2%. e) The statistics of photoelectric performance parameters (*V*_OC_, *J*_SC_, FF, and PCE) distribution for 20 devices. f) PCE distribution of SCs in different seasons (the average temperature/relative humidity (RH) in the laboratory for spring, summer, autumn, and winter are ≈20 °C/≈30% RH, ≈35 °C/≈65% RH, ≈25 °C/≈40% RH, and ≈15 °C/≈15% RH, respectively).

The high‐intensity absorption over a wide range, unique electrical properties, good solution processability, and high stability make the SC‐2D AG‐CQR film ideal candidates for the development of next‐generation SCs. This film (≈150 nm thick) displayed a smooth and uniform surface morphology under ambient conditions (Figure S10, Supporting Information). When exposed to vacuum and air cycles, the absorption intensity of the film showed almost no change under a nitrogen/oxygen atmosphere (<5%) (Figure S11a, Supporting Information). For the 295/375 K annealing temperature cycles, only a slight decrease in intensity was observed (<10%), further indicating the high stability of this film (Figure S11b, Supporting Information). The SCs device structure was composed of an ITO glass substrate anode, a PEDOT:PSS hole injection layer, a SC‐2D AG‐CQR film active layer, a poly[(9,9‐bis(6′‐(N,N,N‐trimethylammonium)hexyl)‐2,7‐fluorene)‐alt‐2,7‐(9,9‐dioctylfluorene)] (PFN‐Br)/Al cathode (**Figure** **4**a), and the corresponding cross‐sectional TEM images are shown in Figure 4b and Figure S12 (Supporting Information). As depicted in the energy diagram of the device, the *E*_HOMO_ of PEDOT:PSS and the *E*_LUMO_ of PFN‐Br matched those of the SC‐2D AG‐CQR film (Figure S5, Supporting Information), showing a small energy barrier for charge injection between the different electrodes and active layers. The high‐energy‐level match between each layer can facilitated charge transfer and the collection of electrons and holes in the electrode.

#### 2.5.2 Device Characterization and Performance

Figure 4b shows the *J*–*V* curves of champion SC‐2D AG‐CQR film‐based SCs measured under simulated AM1.5 illumination of 100 mW cm^−2^. The coincident *J*–*V* between the forward and reverse scans showed low hysteresis and discrepancy, which was a benefit of extremely uniform and dense films. With the SC‐2D AG‐CQR film as the active layer, an open‐circuit voltage (*V*_OC_) of 0.59 V, a short‐circuit current density (*J*_SC_) of 5.35 mA cm^−2^, a fill factor (FF) of 38.64%, and a maximum PCE of 1.22% was recorded. The detailed parameters are listed in **Table** **1**. The external quantum efficiency (EQE) spectra are shown. Under different irradiation times, the value of the EQE and the range of absorbed light were constant, which further showed the low energy loss induced by non‐radiative recombination from the electronic coupling of the SC‐2D AG‐CQR film in SCs (Figure 4c).

**Table 1:** Key optical parameters of SC‐2D AG‐CQR film for SCs.

	*V*_OC_ [V]	*J*_SC_ [mA cm^−2^]	FF [%]	PCE [%]
Forward scan	0.59	5.35	38.64	1.22
Reverse scan	0.59	5.34	37.94	1.21

The operational stability of the unencapsulated/encapsulated devices (1 sun irradiation and stored in a nitrogen atmosphere) is displayed in Figure 4d. The PCE of unencapsulated devices decayed from 1.22% to 0.56% after 120 h of continuous irradiation at 25 °C, retaining ≈50% of the initial value. Additionally, the PCE of the device with encapsulation was excellent after the long‐term continuous irradiation for 380 h at 25 °C, and could maintain ≈50% (≈0.63%) of its initial value (≈1.27%) after continuous irradiation for 270 h at 25 °C. The slow decay of the device, even when exposed to air, indicated the high device stability. The device stability based on SC‐2D AG‐CQRs is significantly higher than that of earlier studies on SCs based on organic materials and perovskite materials.^[^ ^37^, ^38^ ^]^ According to the degradation mechanisms of SCs during operation, a highly uniform film with ideal molecular stacking and fewer surface and internal defects can significantly reduce the number of degradation sites in the initial state and suppress exciton loss and defect migration, which prolongs the operational lifetime of the corresponding devices.

High repeatability of the functional layer fabrication is one of the basic requirements for the commercialization of SCs. To investigate the operational repeatability of the SC‐2D AG‐CQR film‐based SCs, the distribution of the performance parameters of the 20 devices are shown in Figure 4e. On average, the PCE, FF, *J*_SC_, and *V*_OC_ of the devices exhibited a slight change from the average value, and the narrow distribution suggested high device reproducibility of the SC‐2D AG‐CQR film‐based SCs, indicating the potential for development in practical applications.

Owing to the highly crystalline AG‐CQRs structure and the orderly and dense stacking between the AG‐CQRs, the SC‐2D AG‐CQR film exhibited high stability (Figure S11, Supporting Information). Instead of the laborious and expensive control of the annealing atmosphere and different water‐oxygen levels, this film, as an active layer in SCs, is less dependent on the operational climate. The oxygen stability test (1 sun irradiation and storage in a nitrogen/oxygen atmosphere) showed that the devices retained 92% of their initial PCE after 20 cycles (Figure S13a, Supporting Information). Furthermore, thermal stability test (1 sun irradiation and storage at 85 °C in nitrogen atmosphere) was performed. The devices retained 85% of their initial PCE after 20 cycles (Figure S13b, Supporting Information). We also investigated reproducibility under all‐weather in‐house conditions (Figure 4f). Under the condition of four quarters with varied humidity, the SC‐2D AG‐CQR film‐based SCs were clearly independent of weather and had a small performance difference (average PCE of >1.0%) and high reproducibility. This effectively blocked the interference from ambient conditions and all‐weather adaptability for the widespread fabrication of devices. The SC‐2D AG‐CQR film exhibits significant potential for improving the device efficiency and stability, which pioneers research on carbon nanomaterials as active layers in SCs.

## 3 Conclusion

We reported the successful fabrication of AG‐CQRs with highly crystalline hexagonal shapes (aspect ratio of ≈ 2:1) modified by carbonyl groups at both ends. This was achieved by judiciously choosing PT with high charge mobility and different active reaction sites on the benzene ring and carbonyl groups as precursors. The unique structure of the AG‐CQRs determined their wide bandgap absorption (440–850 nm) and high *μ*_e_. Furthermore, by controlling the stacking between the AG‐CQRs, an SC‐2D AG‐CQR film was obtained, in which the four stacking modes were shown to have different electrical properties. Among them, AB mode as the most stable stacking configuration in this film possessed a more balanced charge transfer, determining the film with a large *τ*_n_ (≈2.26 µs), fewer *N*_t_ (≈8.85 × 10^15^ cm^−3^), a long *L*_d_ (≈190 nm), and a high and matched *μ*_e_ and *μ*_h_ up to 2.03 × 10^−3^ cm^2^ V^−1^ s^−1^ and 1.82 × 10^−3^ cm^2^ V^−1^ s^−1^, respectively. Moreover, this film showed high stability in the device operational climate, including temperature and water oxygen level. With the active layer alone, the SCs exhibited a maximum PCE of 1.22%, impressive long‐term operational stability of ≈380 h, and repeatability. This is the first time that carbon nanomaterials have been utilized as active layer materials in SCs to achieve such a high device performance. By further designing the structure of carbon nanomaterials, matching with donor or acceptor materials, and optimizing device structures, we anticipate to inspire further research on the application of carbon‐nanomaterials on SCs and facilitate considerably improvements in device performance. Detailed follow‐up work along this line is underway in our laboratory, and will be reported in due course.

## Conflict of Interest

The authors declare no conflict of interest.

## Supporting information

Supporting Information

Supporting Information

Supporting Information

## Data Availability

The data that support the findings of this study are available from the corresponding author upon reasonable request.
