# Supplementary material for: Axially Growing Carbon Quantum Ribbon with 2D Stacking Control for High‐Stability Solar Cell
Source: Adv Sci (Weinh). 2024 Jul 19;11(35):2400817. doi: 10.1002/advs.202400817 (PMC11425258; doi:10.1002/advs.202400817)
Supplement: Supplementary file 1 — Supporting Information [file ADVS-11-2400817-s002.pdf]

SUPPORTING INFORMATION

Axially growing Carbon Quantum Ribbon with Two-dimensional Stacking Control for High-stability Solar Cell

Yuxin Shi[a], Yongshuai Gong[b], Yang Zhang[a], Yunchao Li[a], Xiaohong Li[a], Zhan'ao Tan*[b] and Louzhen Fan*[a]*

Y. Shi, Prof. Y. Zhang, Prof. Y. Li, Prof. X. Li, Prof. L. Fan
College of Chemistry, Key Laboratory of Theoretical & Computational Photochemistry of Ministry of Education
Beijing Normal University
Beijing 100875, China
E-mail: y.zhang@bnu.edu.cn, lzfan@bnu.edu.cn

[b] Y. Gong, Prof. Z. Tan
Beijing Advanced Innovation Center for Soft Matter Science and Engineering Department
Beijing University of Chemical Technology Institution
Beijing 100029, China
E-mail: tanzhanao@mail.buct.edu.cn

Materials and Methods

Synthesis of AG-CQRs: AG-CQRs were synthesized by solvothermal treatment. In the synthetic process of the AG-CQRs, 5,7,12,14-pentacenetetrone (PT) (100 mg) (Tokyo Chemical Industry, Industrial grade, 99% purity) was dissolved in the formamide solution (15 mL) (Sigma-Aldrich, Analytical reagent, 99.8% purity), followed by the concentrated sulfuric acid (1 mL) (Bei Jing Tong Guang, Analytical reagent, 98% purity). The precursor solution was ultrasonically dissolved for 10 min, then heated in an oven with a poly (tetrafluoroethylene) (Teflon)-lined autoclave (25 mL) at 220 °C for 6 h. And then the reactors were cooled to room temperature at ambient conditions, and sulfuric acid was removed by cleaning multiple times using deionized water. The AG-CQRs were further repeatedly separated and purified though silica gel column chromatography. We used petroleum ether and ethyl acetate with different volume ratios as the eluents to obtain pure sample.

Synthesis of SC-2D AG-CQR film: The AG-CQRs chlorobenzene solution was dropped on the surface of the highly oriented pyrolytic graphite substrate, and placed, and heated at 80°C for 15 minutes in a vacuum atmosphere (10^{-4} Pa). Then, after the above substrate was naturally cooled to room temperature, SC-2D AG-CQR film was obtained by rinsing with ethanol at 3500 rpm for 30 s.

Ultraviolet Photoelectron Spectroscopy Measurement: The UPS of SC-2D AG-CQRs thin film was measured with an hv = 21.22 eV, He I source (AXIS ULTRA DLD, Kratos), analysis room vacuum of 3.0×10^{-8} Torr, and the bias voltage of −9 V.

Characterization Method: A Hitachi SU 8010 scanning electron microscope and a Talos F200S transmission electron microscope (TEM) showed the morphologies of the AG-CQRs. XRD pattern was obtained *via* X-ray diffraction with Cu-Kα radiation (XRD, PANalytical X'Pert Pro MPD). XPS was measured *via* an electron spectrometer with 300 W Al Kα radiation from VG Scientific (ESCALab 220i-XL). Raman spectra were obtained *via* laser confocal micro-Raman spectroscopy (LabRAM Aramis). FT-IR spectra were obtained *via* a Thermo Scientific Nicolet 380 spectrometer. A Shimadzu UV-2450 spectrophotometer measured absorption spectra. Temperature-dependent time-resolved decay spectra were measured *via* an FLS980 fluorescence spectrofluorometer from Edinburgh at 77–300 K through a liquid nitrogen cooler. A Nikon camera (D7200) captured the photographs of samples under daylight/UV (ENF-280C/FBE, 8 W, 365 nm).

AC-TEM images characterization. A FEI-Themis Z transmission electron microscope (TEM) was used to investigate the AC-TEM image of the AG-CQRs. Ultrathin carbon film supported by a lacey on a 400 mesh copper grid (product no. 01824, bought from Beijing Xinxing Braim

Technology Co., Ltd) was used to disperse the AG-CQRs. The purified diluted AG-CQRs ethanol solution with 5 μL was dropped on the surface of ultrathin carbon film, and then dried at room temperature. Finally, the AC-TEM image of AG-CQRs samples were measured at 200 KV.

AFM Characterization: An Asylum Research Cypher atom force microscope (AFM) was used in tapping mode to investigate the stacking of the AG-CQRs. A highly oriented pyrolytic graphite (HOPG) substrate was used to form the SC-2D AG-CQR film. We observed the structure using cantilevers with resonant frequencies of 265–410 kHz and oscillation amplitudes less than 1 nm. Arrow NSC15/AL BS probes (purchased from Beijing Xingde Instrument Equipment Co., Ltd) was used for scanning.

To perform the electronic properties, topography, and friction measurements, with the conductive probes coated with Ti/Ir (ASYELEC-01, Asylum Research) under ORCA mode in ambient conditions (20−25 °C, relative humidity 20−30%) is used in AFM. Silicon probes coated with nitrogen-doped diamond (DCP 10, NT-MDT) were also adopted to perform the electronic measurements of SC-2D AG-CQR film. The *I−V* curves were obtained by applying a varied bias voltage at a constant normal force at different locations.

Theoretical calculations: All the energy level and electron cloud distribution of different types of models of AG-CQRs were calculated. For AG-CQRs models, we selected a time-dependent density functional theory (TDDFT) method installed in Gaussian 09 software, and combined the 6-311G* basis set and the functional B3LYP (B3LYP/6-311G*).

For the different stacking between AG-CQRs models, DFT was performed to achieve optimized geometrical and electronic structures with a projector augmented wave (PAW) basis

as implemented in Vienna Ab Initio Simulation Package code. The Perdew-Burke-Ernzerhof (PBE) Generalized Gradient Approximation (GGA) exchange-correlation functional method was adopted. All calculations were performed using the DFT/GGA method. Moreover, van der Waals interaction (DFT-D2 method) was incorporated. The kinetic energy cutoff was 500 eV. Brillouin zone integration was performed on grids *Γ*-centered 1 × 1 × 1 k-points-grids for the structural relaxation. Total energy and all forces on atoms converged to less than 10^{-5} eV and 0.02 eV/Å. The vacuum space of more than 15 Å along the z direction is used to decouple possible periodic interactions.

Device Fabrication and Characterization: The ITO glass substrates were prepared through cleaning ultrasonically three times in isopropyl, alcohol and deionized water, respectively. After drying in an oven at 150 °C for 15 min, the cleaned substrates were treated with UV–ozone for 15 min to reduce the surface resistance and obtained the ITO with high work function. The poly (3,4-ethylenedioxythiophene):poly (styrenesulfonate) (PEDOT:PSS) was spin coated at 4000 rpm for 35 s on the ITO substrate to prepare a hole injection layer. And after annealed in an oven at 150 °C for 15 min, the thickness is approximately 30 nm. Next, on the surface of the PEDOT:PSS film, the SC-2D AG-CQR film was formed as the active layer. And on the surface of the SC-2D AG-CQR film, the poly[(9,9-bis(6'-(N,N,N-trimethylammonium)hexyl)-2,7-fluorene)-alt-2,7-(9,9-dioctylfluorene)] (PFN-Br) dissolved in methanol was spin coated at 3500 rpm for 35 s to form the interlayer layer of cathode. After that, by putted into the vacuum evaporation chamber, the cathode Al with a thickness of 100 nm (33 Å s^{-1}) were thermally deposited at the pressure of 3×10^{-4} Pa. At the end, the devices have the active area of 4 mm^2 at the overlapping area of Al. The HIL PEDOT:PSS as a buffer layer was mainly used to adjust the energy level of the anode ITO from 4.7 to 5.0 eV and to stabilize the transmission and

injection of holes in the device by reducing the surface roughness and pinhole of the anode/active layer. The interlayer PFN-Br was used to transfer electron and regulate the interaction of phase interfaces between cathode and the active layer. Energy level matching between layers in devices was the prerequisite for the selection of materials. The *J–V* curve was recorded using a solar simulator (SS-F5-3A, Enlitech) along with AM 1.5G spectra whose intensity was calibrated by using a certified standard silicon solar cell (SRC-2020, Enlitech) at 100 mW cm^{-2}. The external quantum efficiency (EQE) was measured using a solar-cell spectral-response measurement system (QE-R, Enlitech). The stability tests were conducted by ageing devices under 1-sun illumination (white LED source, 100 mW cm^{-2}) with constant 0.9 V bias voltage at averaged temperature about 26 ℃.

Supporting Figures

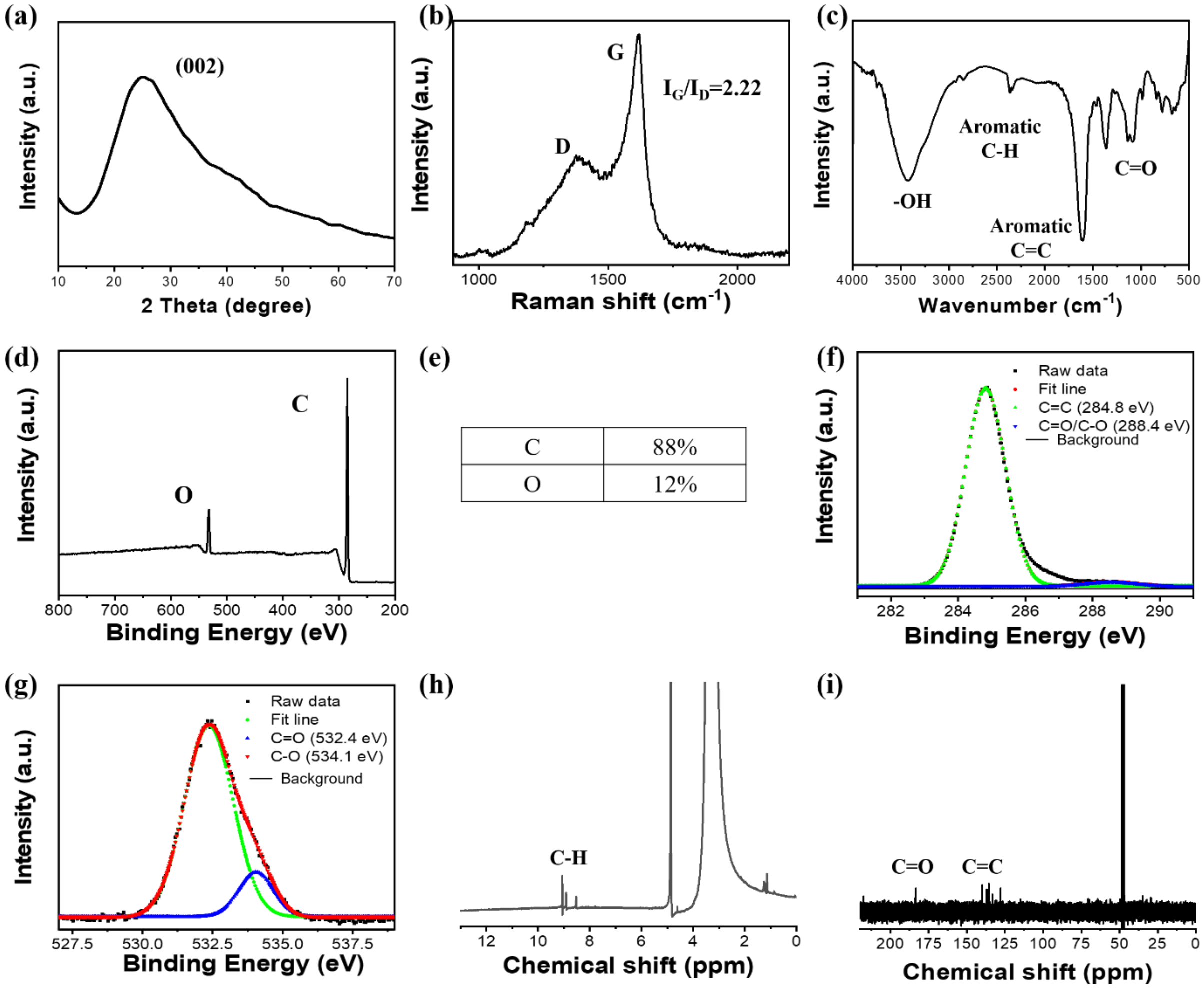

Figure S1.

a) The Raman, b) XRD, c) FT-IR, d) XPS spectra, e) the relative contents of C and O atoms, high-resolution f) C1s and g) O1s, h) ^{1}H-NMR and i) ^{13}C-NMR images of the AG-CQRs.

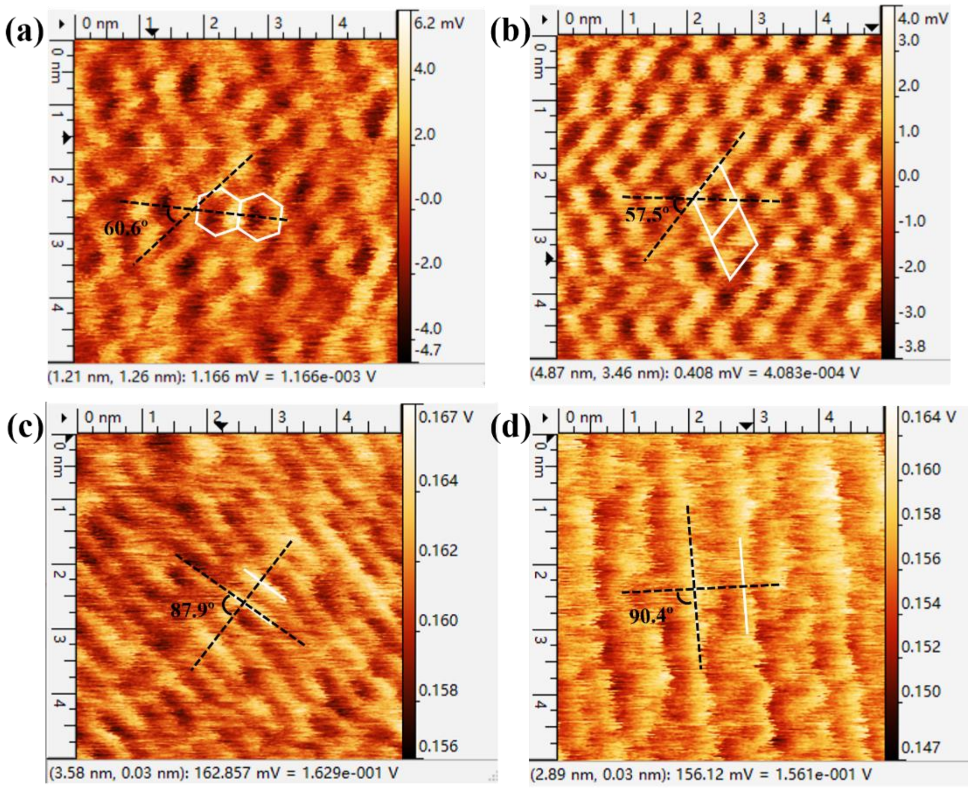

Figure S2.

AFM images of the SC-2D AG-CQR film with four stacking modes, including a) AB, b) AA, c) SP-1 and d) SP-2.

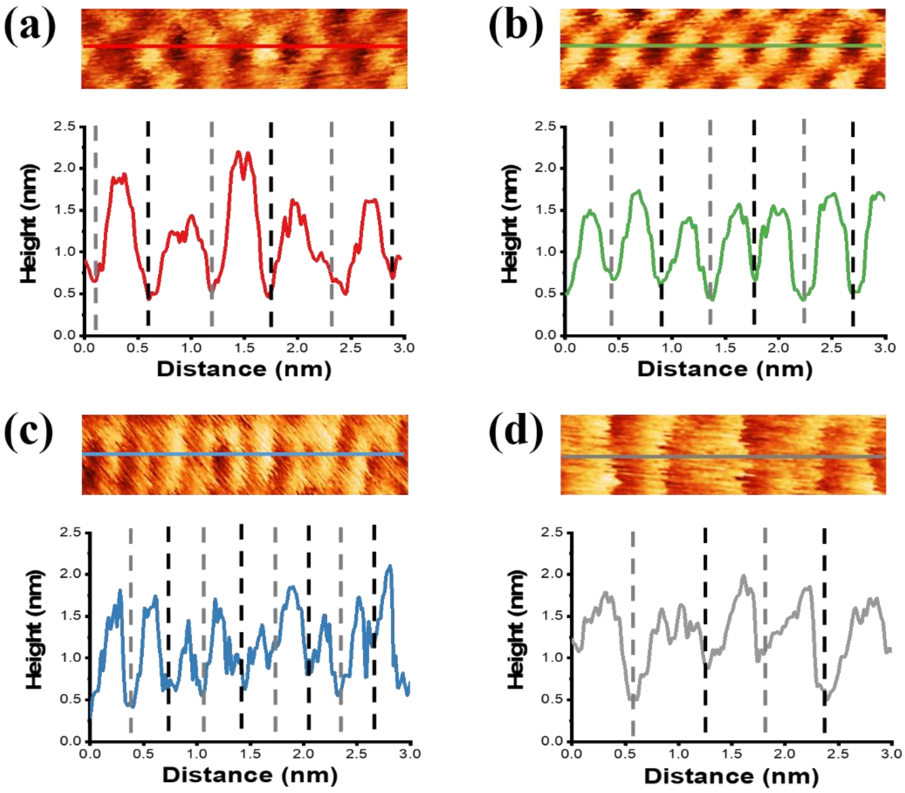

Figure S3.

Height curves of the SC-2D AG-CQR film with four stacking modes, including a) AB, b) AA, c) SP-1 and d) SP-2.

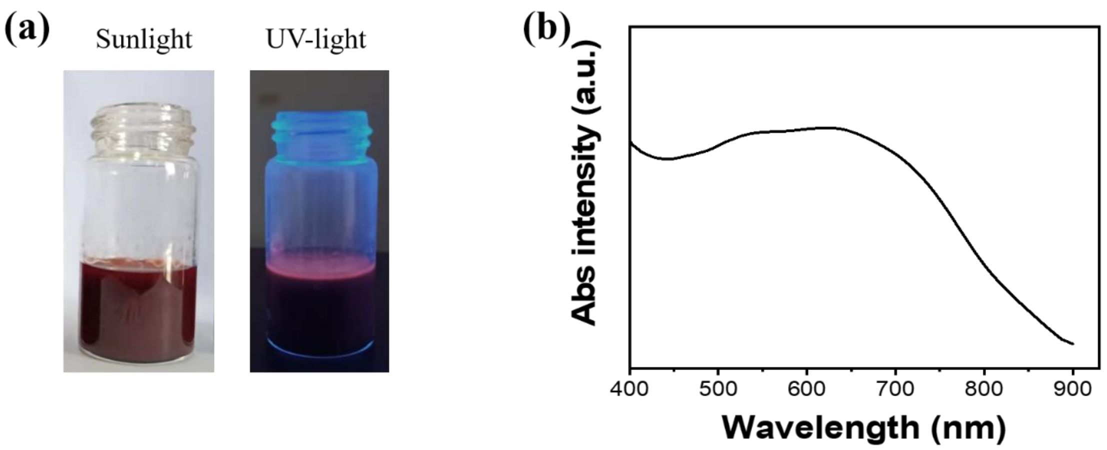

Figure S4.

a) The optical photographs under sunlight and UV light (365 nm) and b) absorption spectrum of the AG-CQRs solution.

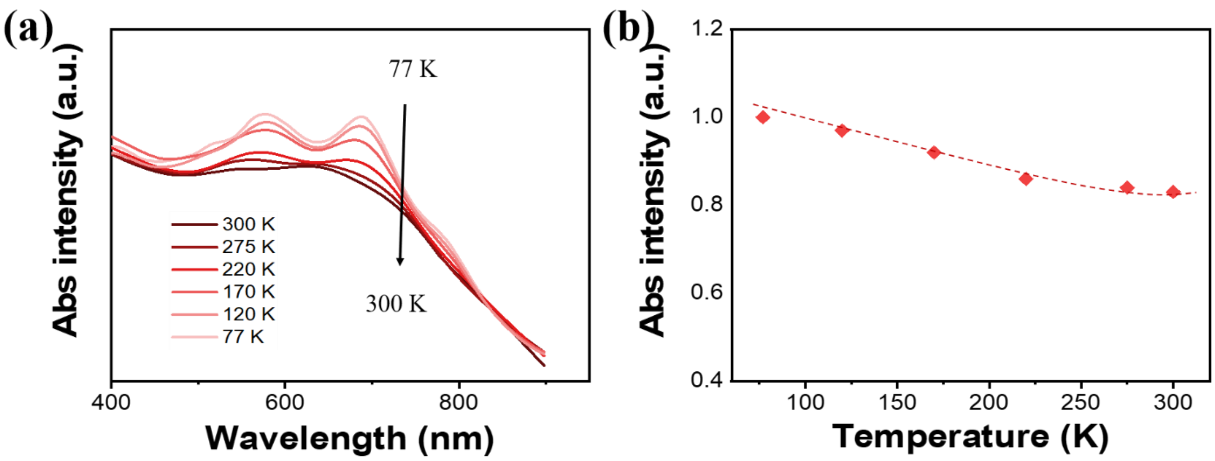

Figure S5.
a) Temperature-dependent UV–vis absorption spectra (77-300 K) and b) the change trend of the SC-2D AG-CQR film.

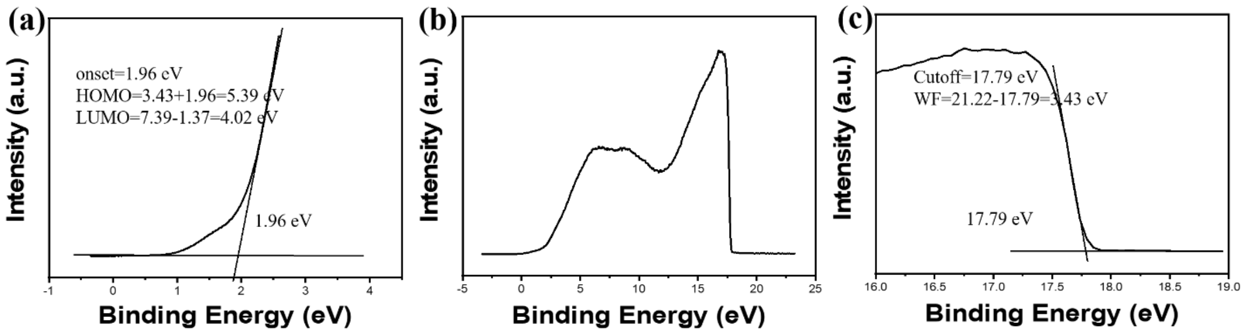

Figure S6.
UPS data of SC-2D AG-CQR film. The work function (WF) is calculated by WF = $h\nu - E_{cutoff}$ (He I, $h\nu$ = 21.22 eV) and the highest occupied molecular orbital (HOMO) is calculated by HOMO = $h\nu - (E_{cutoff} - E_{onset})$. The WF, HOMO, and the lowest unoccupied molecular orbital (LUMO) are calculated to be 3.43, 5.39, and 4.02 eV, respectively.

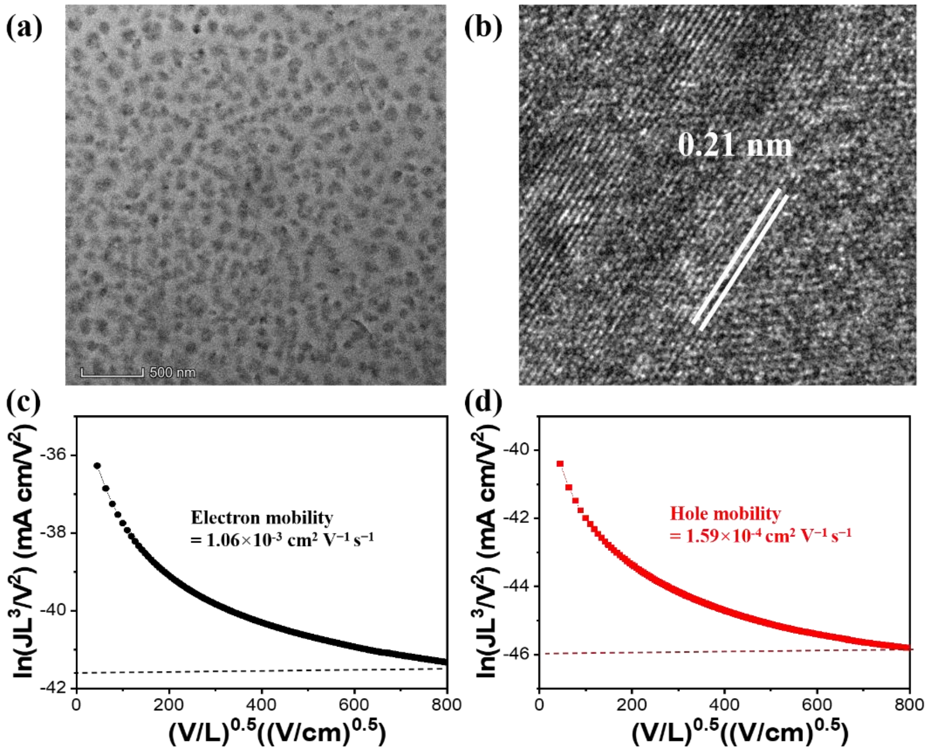

Figure S7.

a) TEM image and b) the corresponding high-resolution TEM image. c)The electron mobility curve of electron-only ITO/SnO_2/active layer/Al device and d) the hole mobility curve of hole-only ITO/PEDOT:PSS/active layer/MnO_3/Al devices. The active layer was prepared by spin-coating the AG-CQRs with 3000 rpm and annealing temperature at 100 °C for 10 min.

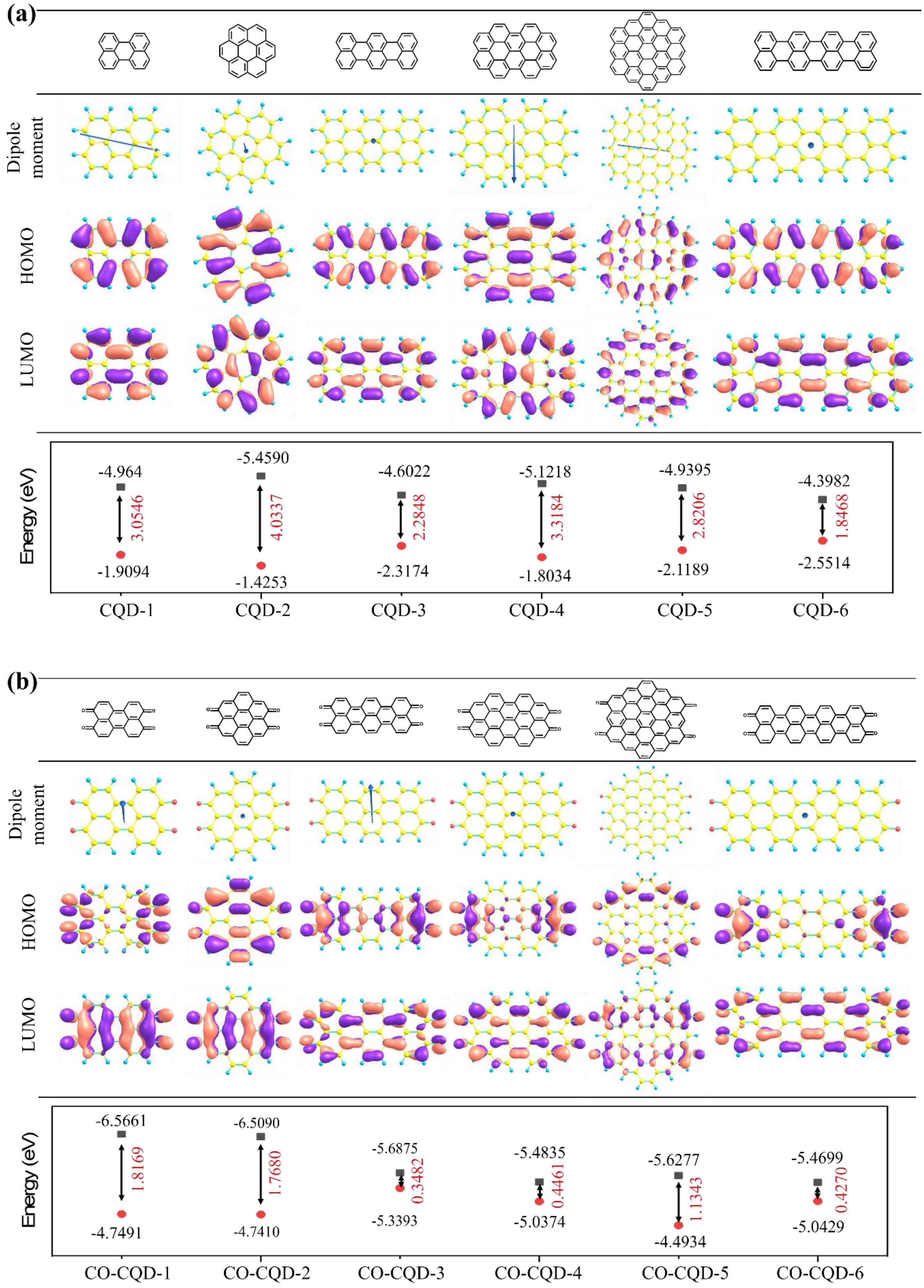
(a)
Dipole moment
HOMO
LUMO
Energy (eV)
-4.964
3.0546
-1.9094
-5.4590
4.0337
-1.4253
-4.6022
2.2848
-2.3174
-5.1218
3.3184
-1.8034
-4.9395
2.8206
-2.1189
-4.3982
1.8468
-2.5514
CQD-1
CQD-2
CQD-3
CQD-4
CQD-5
CQD-6
(b)
Dipole moment
HOMO
LUMO
Energy (eV)
-6.5661
1.8169
-4.7491
-6.5090
1.7680
-4.7410
-5.6875
0.3482
-5.3393
-5.4835
0.4461
-5.0374
-5.6277
1.1343
-4.4934
-5.4699
0.4270
-5.0429
CO-CQD-1
CO-CQD-2
CO-CQD-3
CO-CQD-4
CO-CQD-5
CO-CQD-6

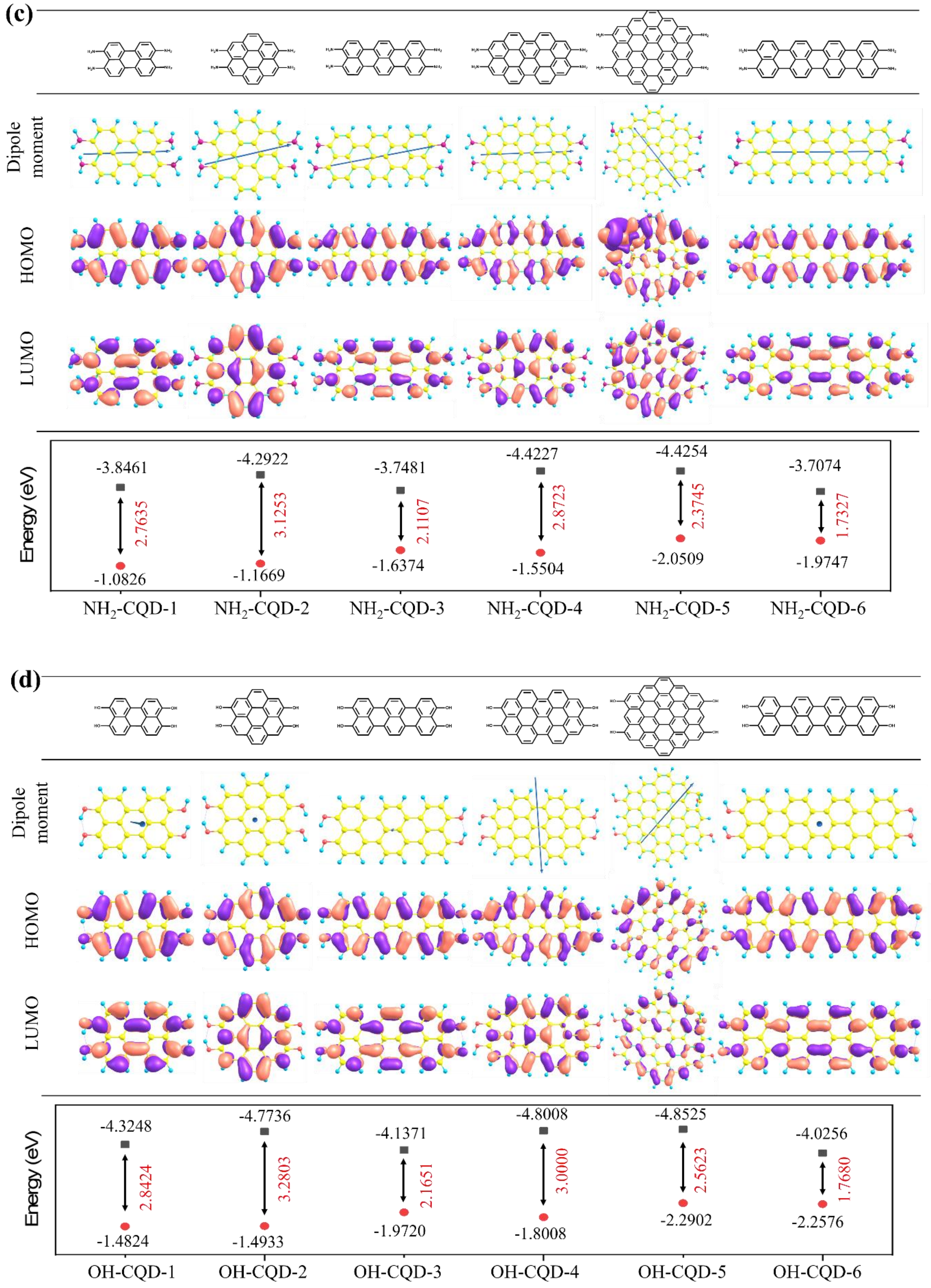
(c)
Dipole moment
HOMO
LUMO
Energy (eV)
-3.8461
2.7635
-1.0826
-4.2922
3.1253
-1.1669
-3.7481
2.1107
-1.6374
-4.4227
2.8723
-1.5504
-4.4254
2.3745
-2.0509
-3.7074
1.7327
-1.9747
NH_2-CQD-1
NH_2-CQD-2
NH_2-CQD-3
NH_2-CQD-4
NH_2-CQD-5
NH_2-CQD-6
(d)
Dipole moment
HOMO
LUMO
Energy (eV)
-4.3248
2.8424
-1.4824
-4.7736
3.2803
-1.4933
-4.1371
2.1651
-1.9720
-4.8008
3.0000
-1.8008
-4.8525
2.5623
-2.2902
-4.0256
1.7680
-2.2576
OH-CQD-1
OH-CQD-2
OH-CQD-3
OH-CQD-4
OH-CQD-5
OH-CQD-6

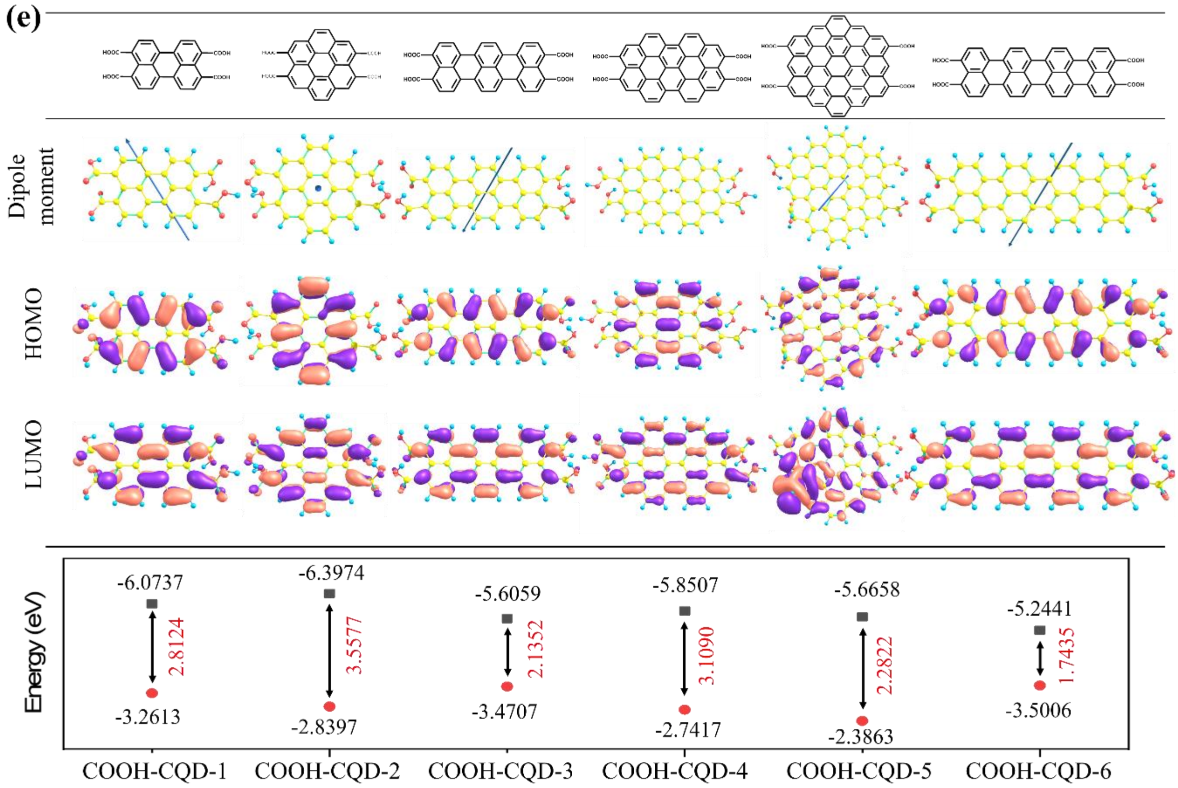

Figure S8.

Structural models, dipole moment, HOMO and LUMO and energy level of AG-CQRs with different aspect ratios from 2:1, 1:1, 3:1, to 4:1, including AG-CQRs a) without modification and modified by b) carbonyl, c) amino, d) hydroxy, and e) carboxyl groups.

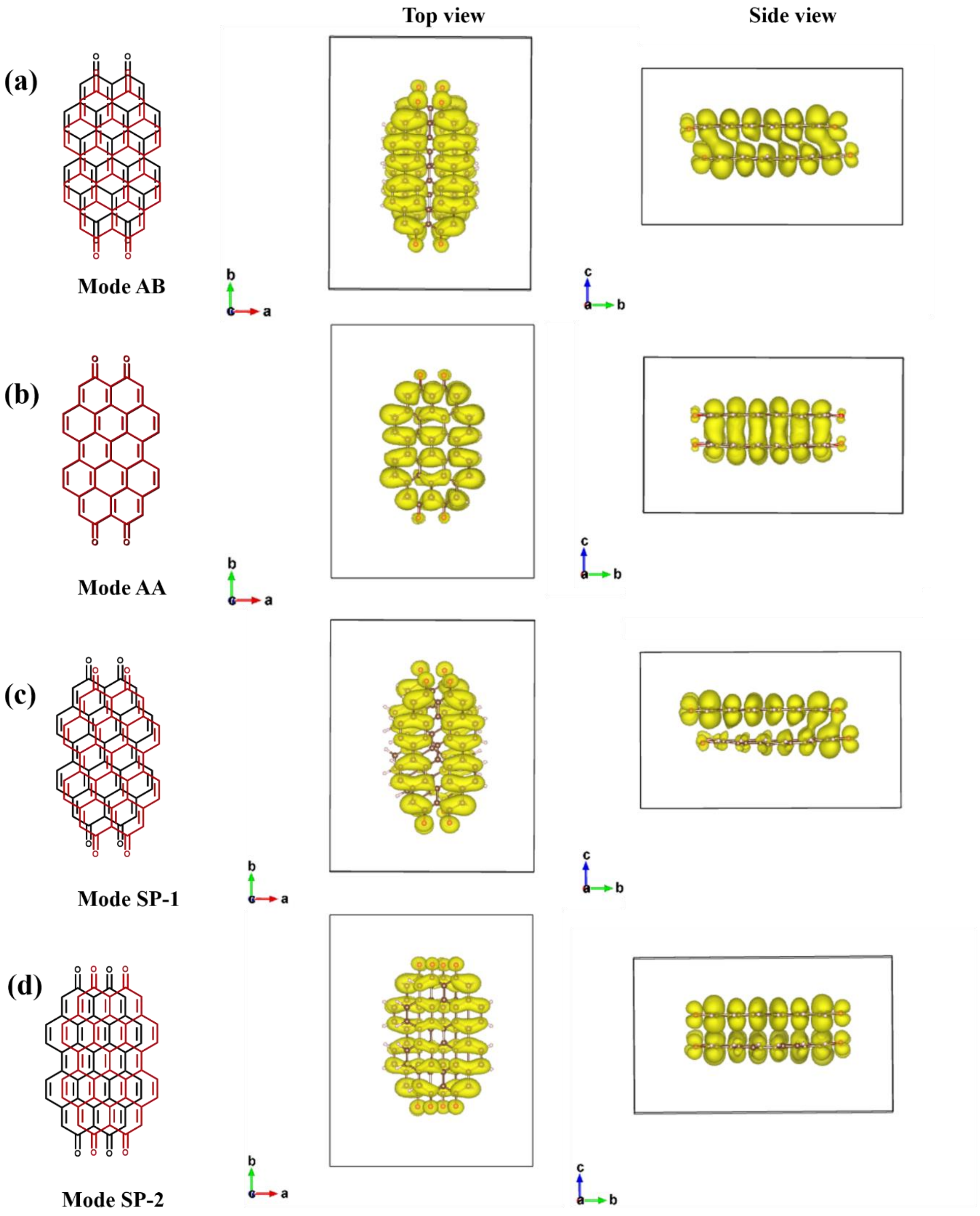

Figure S9.
Structural models and 3D electronic occupation of AG-CQR-AG-CQR with different stacking mode, including a) AB, b) AA, c) SP-1 and d) SP-2 in top view and in side view.

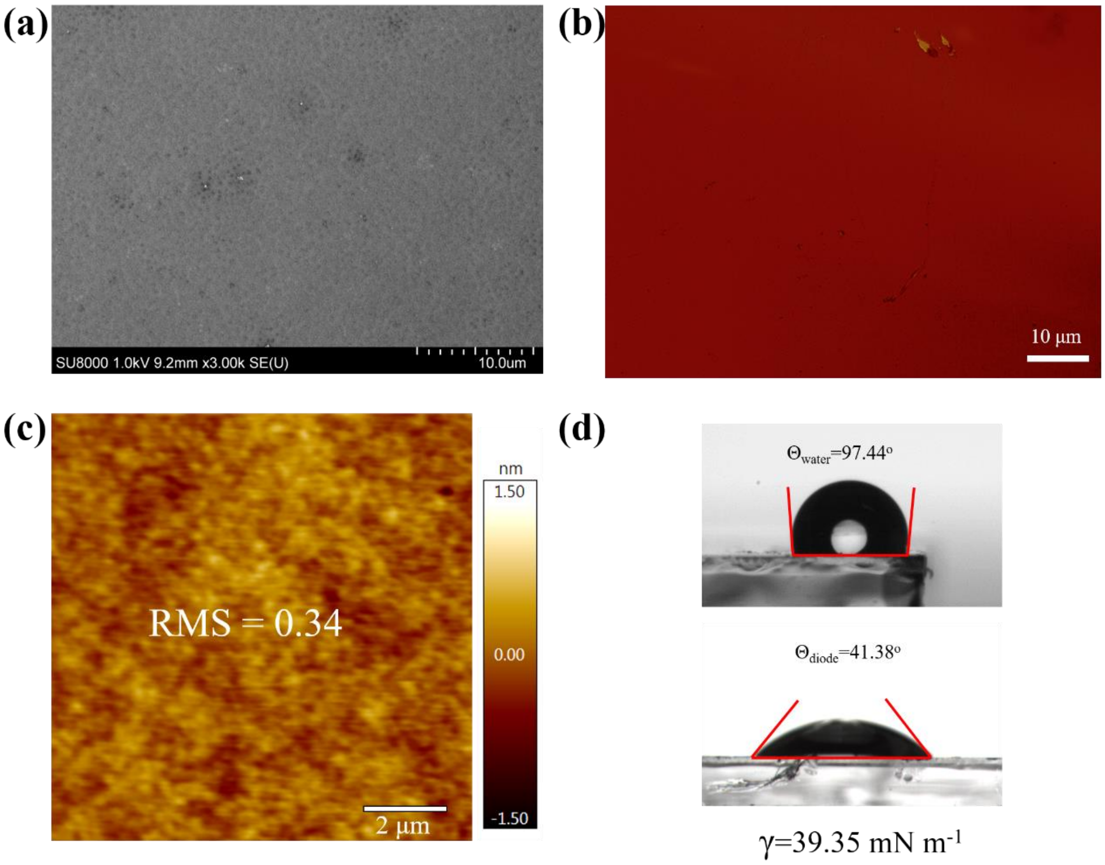

Figure S10.
a) SEM image, b) fluorescence microscope image, c) AFM image and d) the contact angles of water and diiodomethane of the film and the surface free energy (γ) calculated from the contact angle results according to the Owens–Wendt method.

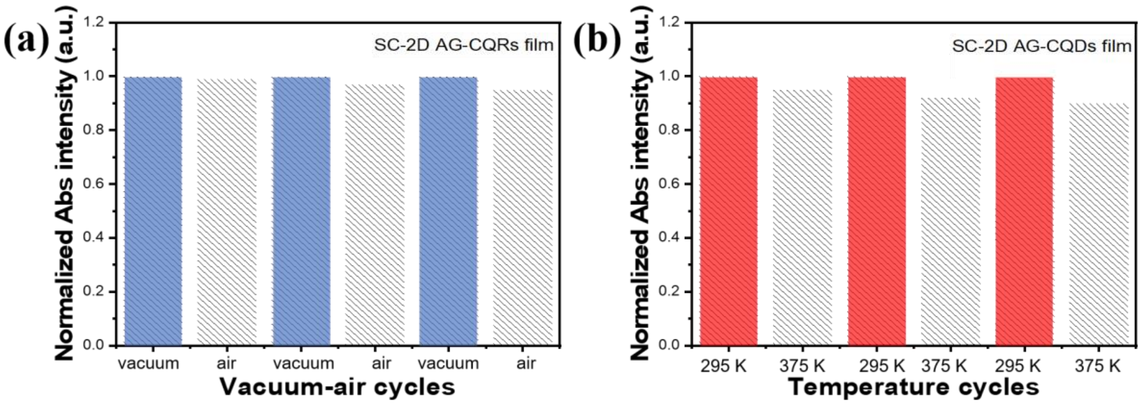

Figure S11.

a) Reversible Abs quenching by exposure to oxygen of SC-2D AG-CQR films. Change in Abs intensity of a thin film under sequential vacuum-air cycles, normalized to the intensity of the first measurement. (Vacuum of 10^{-5}mBar in the glove box) Samples stabilized for 0.5 h under vacuum or in air prior to each measurement. b) Reversible Abs quenching of SC-2D AG-CQR films for temperature. Change in Abs intensity of a thin film under sequential temperature cycles, normalized to the intensity of the first measurement. Samples stabilized for 0.5 h under 295 K or 375 K prior to each measurement.

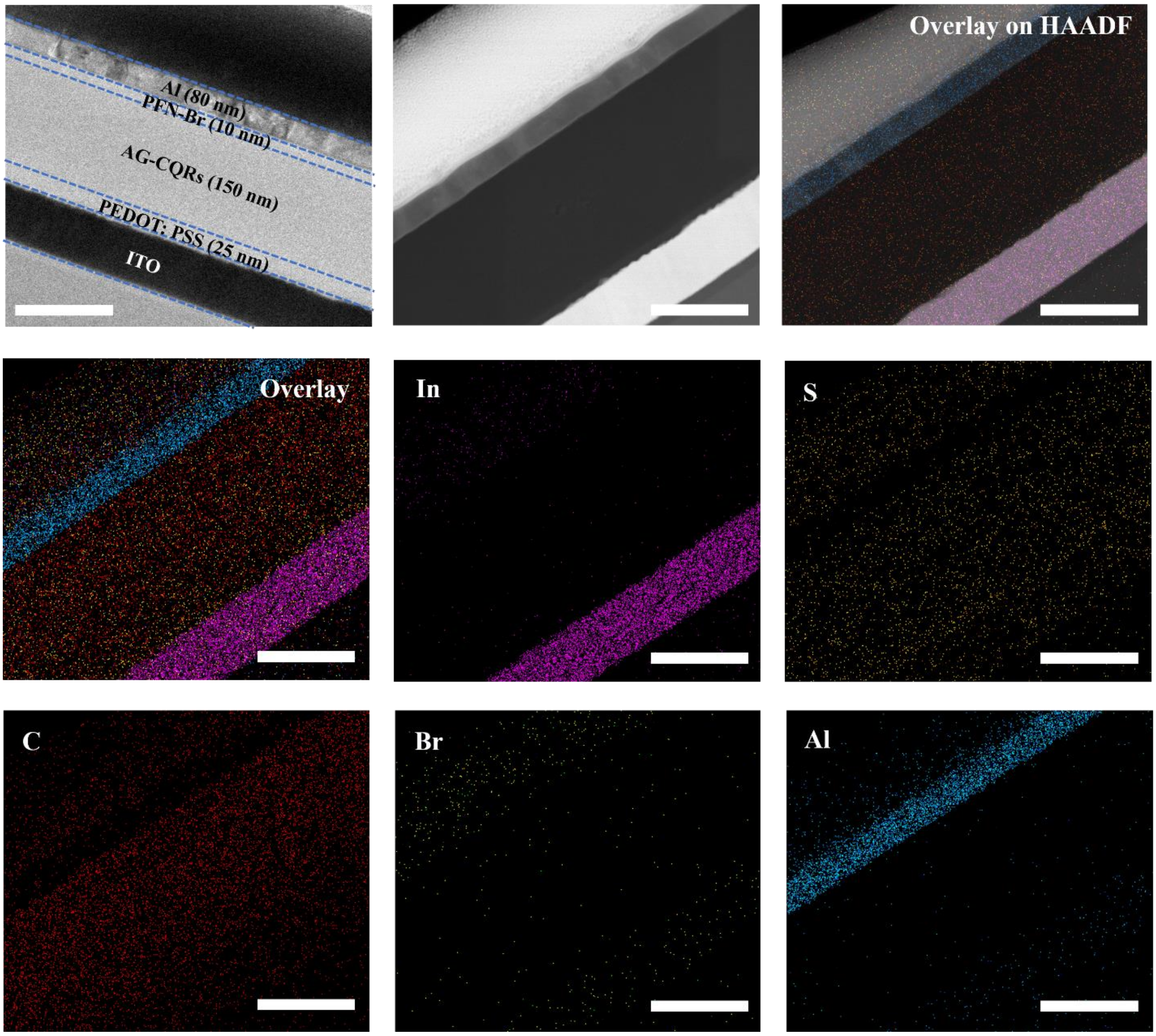

Figure S12.

The cross-sectional TEM images and corresponding EDX mapping images of SCs (Scale bar: 50 nm).

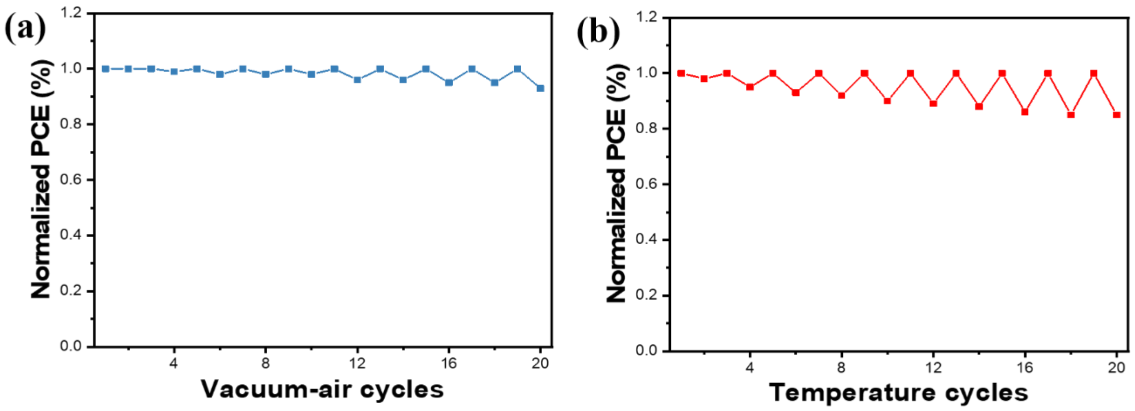

Figure S13.
a) Reversible PCE by exposure to oxygen of SCs under sequential vacuum-air cycles by stabilized for 20 cycles. Change in PCE of a SC under sequential vacuum-air cycles, normalized to the PCE value of the first measurement. (Vacuum of 10^{-5} mBar in the glove box). b) Reversible PCE of SCs under sequential 295-375 K temperature cycles by stabilized for 20 cycles. Change in PCE of SCs under sequential temperature cycles, normalized to the PCE value of the first measurement.

Supporting Tables

Table S1. Key parameters of some typical materials reported for SC applications.

Materials categories	Molecules	λ_{abs} (nm)	L_D (nm)	μ (cm^2 V^{-1} s^{-1})	Ref.
Organic materials	Pentacene/C_{60}	--	65/40	--	[1]
	DRCN7T:PC_{71}BM/ DERHD7T:PC_{71}BM	--	--	5.91×10^{-4}/ 1.18×10^{-4}	[2]
	P3HT	330-650	8.5	--	[3]
	PBDB-TF:eC9/ PBDB-TF:HDO-4Cl:eC9/ PBDB-TF: HDO-4Cl	~420-930	-- /12.2/16.3	1.6×10^{-4}/2.0×10^{-4}/1.3×10^{-4}	[4]
	PM6:Y6	~420-700	65.4	1.4×10^{-3}/ 1.1×10^{-3}	[5]
	PM6:QX-1/QX-2	~450-800	9.5/12.6	3.64×10^{-4}/ 3.92×10^{-4}	[6]
	BTP-2FCl/L8-BO	~550-950	7.02/4.98	8.8×10^{-4}/2.0×10^{-4}/5.09×10^{-4}	[7]

Carbon nanomaterials	AG-CQRs	~440-850	190	2.03×10^{-3}/ 1.82×10^{-3}	In this work

Table S2.
Optimized Cartesian coordinates (Å) of AG-CQRs-1 without the modification for the ground state.

C	0.7382252	1.2498938	-2.16E-05
C	-0.7382252	1.2498937	1.12E-05
C	-1.4392515	0	4.7E-06
C	-0.7382252	-1.2498938	-5.3E-06
C	0.7382252	-1.2498937	1.57E-05
C	1.4392515	0	-4.7E-06
C	-2.8744635	0	9.9E-06
C	-3.5751301	-1.2326937	-1.11E-05
C	2.8854469	-2.4225014	5.62E-05
C	-1.4792556	-2.4274788	-3.24E-05
C	-1.4792556	2.4274789	4.72E-05
C	-2.8854468	2.4225015	5.98E-05
C	-3.5751301	1.2326936	3.68E-05
C	2.8744635	0	-9.9E-06
C	3.5751301	1.2326937	-4.71E-05
C	2.8854469	2.4225014	-8.0E-05
C	1.4792556	2.4274788	-6.74E-05
C	1.4792557	-2.4274789	5.26E-05
C	-2.8854469	-2.4225014	-3.6E-05
C	3.5751301	-1.2326936	2.14E-05
H	-4.6614575	-1.2182595	-8.9E-06
H	3.4206316	-3.3674407	8.51E-05
H	-0.9764906	-3.3872976	-5.34E-05
H	-0.9764906	3.3872975	6.89E-05
H	-3.4206316	3.3674407	8.66E-05
H	-4.6614575	1.2182596	4.16E-05
H	4.6614575	1.2182595	-5.18E-05
H	3.4206316	3.3674407	-0.0001148
H	0.9764906	3.3872976	-9.72E-05
H	0.9764906	-3.3872976	8.16E-05
H	-3.4206316	-3.3674407	-5.69E-05
H	4.6614575	-1.2182595	1.92E-05

Optimized Cartesian coordinates (Å) of AG-CQRs-1 modified by carbonyl group for the ground state.

C	0.6948216	1.2586159	0.0002667
C	-0.6948218	1.2586159	-0.000133
C	-1.4448865	-2.2E-06	2.7E-06
C	-0.694823	-1.2586215	0.0001464
C	0.6948225	-1.2586215	-0.0002484
C	1.4448863	-2.4E-06	5.0E-06
C	-2.8319617	0	-3.1E-06
C	-3.5967557	-1.2863703	0.000703
C	-2.7963754	-2.5172961	0.0009134
C	-1.4516358	-2.4972292	0.0006484
C	-1.4516291	2.4972264	-0.0006352
C	-2.7963686	2.5172967	-0.0009121
C	-3.5967518	1.2863728	-0.0007151
C	2.8319618	-1.0E-07	-2.3E-06
C	3.596752	1.286373	0.0005014
C	2.7963686	2.5172968	0.001086
C	1.4516289	2.4972263	0.0009344
C	1.4516355	-2.4972292	-0.000901
C	2.7963753	-2.5172961	-0.0010607
C	3.5967556	-1.2863706	-0.0005138
O	4.8210629	1.3523499	0.0004998
O	-4.8210664	-1.352343	0.0012579
O	-4.8210623	1.3523492	-0.0012856
O	4.8210668	-1.3523439	-0.0005478
H	-3.3597396	-3.4443735	0.0014526
H	-0.9222978	-3.4400746	0.0010339
H	-0.9222858	3.4400698	-0.001014
H	-3.3597299	3.4443759	-0.0014534
H	3.3597301	3.444376	0.0016609
H	0.9222856	3.4400695	0.0014998
H	0.9222976	-3.4400746	-0.0014458
H	3.3597393	-3.4443737	-0.0016245

Optimized Cartesian coordinates (Å) of AG-CQRs-1 modified by amino group for the ground state.

C	-0.7340328	-1.2337931	-0.0600626
C	0.734026	-1.2337894	0.0601195
C	1.4588126	1.0E-07	1.0E-07
C	0.734026	1.2337894	-0.0601195
C	-0.7340328	1.2337931	0.0600626
C	-1.4588149	1.0E-07	-1.0E-07

C	-0.7340328	-1.2337931	-0.0600626
C	2.9022856	0	0
C	3.5869899	1.2445409	-0.2189586
C	-2.8487603	2.4131172	0.3228977
C	1.4553907	2.4096191	-0.2253611
C	1.4553906	-2.409619	0.225361
C	2.8487495	-2.4131079	0.3229734
C	3.5869898	-1.2445409	0.2189586
C	-2.9022862	0	0
C	-3.5869837	-1.2445301	-0.2190162
C	-2.8487603	-2.4131172	-0.3228977
C	-1.455403	-2.4096295	-0.2252401
C	-1.455403	2.4096295	0.2252403
C	2.8487495	2.413108	-0.3229734
C	-3.5869839	1.2445301	0.2190162
N	-4.9917373	-1.3124707	-0.3023054
N	-4.9917371	1.312471	0.3023055
N	4.991741	-1.3125061	0.3020266
N	4.9917409	1.3125064	-0.3020267
H	-3.3687305	3.3519045	0.4993793
H	0.93999	3.3583022	-0.3219382
H	0.93999	-3.3583022	0.321938
H	3.3687195	-3.3518919	0.4994763
H	-3.3687305	-3.3519044	-0.4993795
H	-0.9400072	-3.3583233	-0.3217384
H	-0.9400072	3.3583233	0.3217387
H	3.3687196	3.351892	-0.4994761
H	-5.4058381	-0.5581671	-0.8385386
H	-5.3080941	-2.2130748	-0.6409628
H	-5.4058388	0.5581644	0.8385338
H	-5.3080941	2.2130735	0.6409667
H	5.4059282	-0.5583989	0.8384665
H	5.3081583	-2.21325	0.6402505
H	5.3081581	2.2132488	-0.6402545
H	5.4059288	0.5583964	-0.8384621

Optimized Cartesian coordinates (Å) of AG-CQRs-1 modified by hydroxy group for the ground state.

C	-0.7439932	1.2386444	0.0004207
C	0.7306384	1.2450185	-0.0004226
C	1.4517375	0.0105582	0.0001395
C	0.7439846	-1.2386434	0.0002651

C	-0.7306377	-1.2450125	-0.0002486
C	-1.4517408	-0.0105538	-8.72E-05
C	2.8942061	0.0219619	0.0001981
C	3.5742429	-1.2306333	0.0008808
C	-2.8560777	-2.4539779	-0.0011125
C	1.4806145	-2.4161425	0.0006419
C	1.4615275	2.4311147	-0.0018951
C	2.8560796	2.453977	-0.0020007
C	3.5841342	1.2770132	-0.0007455
C	-2.8942153	-0.0219564	-0.0001147
C	-3.5742408	1.2306373	0.0007649
C	-2.8827145	2.4185568	0.0019195
C	-1.4806164	2.4161455	0.0018109
C	-1.4615288	-2.4311128	-0.0006869
C	2.8827151	-2.4185485	0.0010847
C	-3.5841318	-1.2770167	-0.0008807
O	-4.9567017	1.2102855	0.0007038
O	-4.9351336	-1.3934421	-0.0013448
O	4.9351345	1.3934113	-0.0006992
O	4.9567106	-1.2102752	0.0013773
H	-3.3991593	-3.3927269	-0.0015711
H	0.9820396	-3.3772892	0.0006225
H	0.9506941	3.386607	-0.0032263
H	3.3991602	3.3927251	-0.0031006
H	-3.4231215	3.3626507	0.0029696
H	-0.9820333	3.3772874	0.0030735
H	-0.9506922	-3.3866033	-0.0007035
H	3.4231258	-3.3626414	0.0015372
H	-5.2980885	2.1133248	0.001075
H	-5.3326496	-0.50679	-0.0010067
H	5.3326511	0.5067624	-0.000129
H	5.2980934	-2.1133198	0.000571

Optimized Cartesian coordinates (Å) of AG-CQRs-1 modified by carboxyl group for the ground state.

C	0.7315907	-1.2373113	0.0625997
C	-0.7418362	-1.2376066	0.001376
C	-1.4587175	0.0037908	-0.0027179
C	-0.7397386	1.2439352	-0.0027305
C	0.7332521	1.2418116	-0.066635
C	1.4485565	0.001855	-0.0018172
C	-2.9017538	0.0059666	-0.0046656

C	0.7315907	-1.2373113	0.0625997
C	-3.576532	1.2532346	0.2017581
C	2.8565128	2.4127235	-0.2835409
C	-1.4609021	2.4307785	0.0553632
C	-1.4636647	-2.4256826	-0.0448816
C	-2.853653	-2.4255137	-0.1740497
C	-3.5780209	-1.2457637	-0.1991804
C	2.8839025	0.0007072	-0.0021726
C	3.5726288	-1.23326	0.2107585
C	2.8529701	-2.4122606	0.2745703
C	1.4558759	-2.4177209	0.1759222
C	1.4593979	2.4205091	-0.1850206
C	-2.8520492	2.4307759	0.1846272
C	3.5751891	1.2332993	-0.2154118
C	5.0220836	-1.2783396	0.6124722
O	5.4834971	-0.5537223	1.463056
O	5.7657693	-2.2626632	0.0600109
C	5.0251087	1.2735536	-0.6136397
O	5.4918886	0.5346682	-1.4492398
O	5.7665206	2.2672318	-0.0746459
C	-5.015956	-1.4130729	-0.5838051
O	-5.6827512	-2.3899603	-0.3143308
O	-5.4704844	-0.4017194	-1.3560832
C	-5.0253113	1.41732	0.5737952
O	-5.7004541	2.354022	0.2247314
O	-5.5341934	0.502443	1.4447767
H	3.3756347	3.3480315	-0.4705646
H	-0.953854	3.3871204	0.0554877
H	-0.9574058	-3.3822043	-0.0379139
H	-3.3905571	-3.3595291	-0.298129
H	3.3716303	-3.3486407	0.4572384
H	0.9508259	-3.3731076	0.2373796
H	0.9549803	3.3758499	-0.2524788
H	-3.3888878	3.3649874	0.3076778
H	5.3255835	-2.5892294	-0.7387487
H	5.3205503	2.6114038	0.7133937
H	-6.4120202	-0.5782184	-1.5239414
H	-4.8484209	-0.1212361	1.7265534

Table S3.

Optimized Cartesian coordinates (Å) of stacking mode AB.

C	7.5442	8.03475	6.520785
C	9.9176	7.95625	6.40371
C	8.7218	7.226	6.522225
C	12.4122	7.96625	6.410025
C	11.1618	7.2335	6.282225
C	6.3352	10.1815	6.512085
C	8.7506	10.13	6.493605
C	7.5262	9.408	6.515925
C	11.1608	10.091	6.251595
C	9.9164	9.375	6.339915
C	13.6602	10.11275	6.410115
C	12.4116	9.375	6.338985
C	7.5876	12.235	6.422565
C	6.3436	11.52575	6.285165
C	9.9854	12.27075	6.3387
C	8.8176	11.52675	6.42786
C	12.4124	12.2335	6.24852
C	11.166	11.52	6.24915
C	13.6604	11.516	6.282165
C	6.3384	14.39875	6.57612
C	8.778	14.39975	6.513315
C	7.5814	13.66375	6.439725
C	11.1648	14.37775	6.25833
C	9.9544	13.669	6.377445
C	13.6596	14.399	6.40806
C	12.4122	13.663	6.251595
C	7.5848	16.5325	6.4452
C	6.3384	15.80325	6.456585
C	9.9966	16.54425	6.57816
C	8.818	15.808	6.582645
C	12.4124	16.524	6.34017
C	11.1676	15.8045	6.41814
C	13.661	15.803	6.341205
C	8.8182	18.76875	6.518505
C	7.5868	17.9505	6.43221
C	11.1772	18.74075	6.68772
C	9.9974	17.94975	6.69378
C	12.4128	17.948	6.43584
C	7.4838	6.55025	9.39954
C	9.857	6.47175	9.552825

C	8.6614	5.74125	9.54711
C	12.3516	6.4815	9.452535
C	11.1014	5.74875	9.37791
C	6.2746	8.697	9.377115
C	8.69	8.6455	9.49065
C	7.4658	7.92325	9.448845
C	11.1004	8.60625	9.552315
C	9.856	7.8915	9.55377
C	13.5998	8.628	9.463725
C	12.351	7.89075	9.502065
C	7.527	10.75025	9.493635
C	6.283	10.041	9.408435
C	9.9248	10.78625	9.47532
C	8.7572	10.04225	9.47778
C	12.3518	10.74875	9.50487
C	11.1056	10.03525	9.508215
C	13.6	10.0315	9.495975
C	6.278	12.914	9.383685
C	8.7174	12.915	9.473025
C	7.5208	12.179	9.426555
C	11.1042	12.893	9.552705
C	9.894	12.18425	9.52251
C	13.5992	12.91425	9.463335
C	12.3516	12.17825	9.505545
C	7.5244	15.04775	9.46035
C	6.278	14.3185	9.429975
C	9.9362	15.0595	9.520185
C	8.7576	14.3235	9.49917
C	12.3518	15.03925	9.553245
C	11.107	14.32	9.553665
C	13.6006	14.31825	9.49827
C	8.7578	17.28425	9.446985
C	7.5264	16.46575	9.37761
C	11.1168	17.256	9.517065
C	9.9368	16.46525	9.553755
C	12.3522	16.46325	9.509865
O	8.6596	5.9335	6.693195
O	11.161	5.977	6.408615
O	8.8388	20.0505	6.52251
O	11.1746	19.999	6.69693
O	8.5992	4.449	9.554115
O	11.1004	4.4925	9.196485
O	8.7784	18.56575	9.37656

O	11.114	18.51425	9.375
H	6.584	7.51925	6.596325
H	13.4064	7.48725	6.503025
H	14.6588	9.6545	6.516975
H	5.4634	12.155	6.567705
H	14.6454	12.03175	6.568455
H	5.3386	13.9465	6.608865
H	14.6576	13.94475	6.51633
H	5.3618	16.3575	6.597045
H	14.6362	16.358	6.567825
H	6.5958	18.48575	6.597165
H	13.403	18.48725	6.596505
H	5.341	9.763	6.431115
H	6.5234	6.0345	9.46173
H	13.3458	6.0025	9.30936
H	14.5984	8.16975	9.33003
H	5.4028	10.67025	9.32823
H	14.585	10.547	9.46476
H	5.2782	12.46175	9.46269
H	14.5972	12.46025	9.330525
H	5.3012	14.873	9.46182
H	14.5758	14.87325	9.45888
H	6.5352	17.001	9.28851
H	13.3426	17.0025	9.464505
H	5.2804	8.27825	9.269595

Optimized Cartesian coordinates (Å) of stacking mode AA.

C	7.5442	8.03475	6.52563
C	9.9176	7.95625	6.424635
C	8.7218	7.226	6.522105
C	12.4122	7.96625	6.43995
C	11.1618	7.2335	6.429825
C	6.3352	10.1815	6.511125
C	8.7506	10.13	6.512355
C	7.5262	9.408	6.514365
C	11.1608	10.091	6.27801
C	9.9164	9.375	6.42165
C	13.6602	10.11275	6.408165
C	12.4116	9.375	6.338925
C	7.5876	12.235	6.42468
C	6.3436	11.52575	6.28395
C	9.9854	12.27075	6.417

C	8.8176	11.52675	6.51138
C	12.4124	12.2335	6.337095
C	11.166	11.52	6.33837
C	13.6604	11.516	6.283125
C	6.3384	14.39875	6.414555
C	8.778	14.39975	6.510855
C	7.5814	13.66375	6.42903
C	11.1648	14.37775	6.255705
C	9.9544	13.669	6.379335
C	13.6596	14.399	6.336345
C	12.4122	13.663	6.25197
C	7.5848	16.5325	6.42594
C	6.3384	15.80325	6.341805
C	9.9966	16.54425	6.576105
C	8.818	15.808	6.58044
C	12.4124	16.524	6.33924
C	11.1676	15.8045	6.416955
C	13.661	15.803	6.28041
C	8.8182	18.76875	6.519375
C	7.5868	17.9505	6.43404
C	11.1772	18.74075	6.68409
C	9.9974	17.94975	6.693
C	12.4128	17.948	6.45756
C	7.4838	8.05025	9.41226
C	9.857	7.97175	9.550125
C	8.6614	7.24125	9.461925
C	12.3516	7.9815	9.375
C	11.1014	7.24875	9.45492
C	6.2746	10.197	9.394395
C	8.69	10.1455	9.382185
C	7.4658	9.42325	9.407535
C	11.1004	10.10625	9.46923
C	9.856	9.3915	9.479355
C	13.5998	10.128	9.46728
C	12.351	9.39075	9.463335
C	7.527	12.25025	9.54516
C	6.283	11.541	9.480915
C	9.9248	12.28625	9.47598
C	8.7572	11.54225	9.43914
C	12.3518	12.24875	9.46449
C	11.1056	11.53525	9.474765
C	13.6	11.5315	9.46692
C	6.278	14.414	9.41811

C	8.7174	14.415	9.454425
C	7.5208	13.679	9.46794
C	11.1042	14.393	9.481485
C	9.894	13.68425	9.471735
C	13.5992	14.41425	9.469065
C	12.3516	13.67825	9.46701
C	7.5244	16.54775	9.451815
C	6.278	15.8185	9.461895
C	9.9362	16.5595	9.51747
C	8.7576	15.8235	9.38541
C	12.3518	16.53925	9.54702
C	11.107	15.82	9.552195
C	13.6006	15.81825	9.47517
C	8.7578	18.78425	9.425505
C	7.5264	17.96575	9.378375
C	11.1168	18.756	9.478635
C	9.9368	17.96525	9.48735
C	12.3522	17.96325	9.378285
O	8.6596	5.9335	6.6924
O	11.161	5.977	6.51759
O	8.8388	20.0505	6.556635
O	11.1746	19.999	6.696885
O	8.5992	5.949	9.375
O	11.1004	5.9925	9.375
O	8.7784	20.06575	9.378075
O	11.114	20.01425	9.553455
H	6.584	7.51925	6.59583
H	13.4064	7.48725	6.50922
H	14.6588	9.6545	6.51213
H	5.4634	12.155	6.56655
H	14.6454	12.03175	6.56766
H	5.3386	13.9465	6.59403
H	14.6576	13.94475	6.487245
H	5.3618	16.3575	6.565695
H	14.6362	16.358	6.54759
H	6.5958	18.48575	6.59322
H	13.403	18.48725	6.595605
H	5.341	9.763	6.43065
H	6.5234	7.5345	9.462255
H	13.3458	7.5025	9.36552
H	14.5984	9.66975	9.456915
H	5.4028	12.17025	9.45678
H	14.585	12.047	9.45135

H	5.2782	13.96175	9.46296
H	14.5972	13.96025	9.45339
H	5.3012	16.373	9.46578
H	14.5758	16.37325	9.45396
H	6.5352	18.501	9.442845
H	13.3426	18.5025	9.30939
H	5.2804	9.77825	9.286065

Optimized Cartesian coordinates (Å) of stacking mode SP-1.

C	7.5442	8.03475	6.518325
C	9.9176	7.95625	6.427635
C	8.7218	7.226	6.522945
C	12.4122	7.96625	6.416205
C	11.1618	7.2335	6.42273
C	6.3352	10.1815	6.51564
C	8.7506	10.13	6.517755
C	7.5262	9.408	6.5139
C	11.1608	10.091	6.256035
C	9.9164	9.375	6.424035
C	13.6602	10.11275	6.266715
C	12.4116	9.375	6.250485
C	7.5876	12.235	6.43335
C	6.3436	11.52575	6.43776
C	9.9854	12.27075	6.510345
C	8.8176	11.52675	6.58089
C	12.4124	12.2335	6.10602
C	11.166	11.52	6.33675
C	13.6604	11.516	6.251505
C	6.3384	14.39875	6.44349
C	8.778	14.39975	6.51504
C	7.5814	13.66375	6.429885
C	11.1648	14.37775	6.277755
C	9.9544	13.669	6.443925
C	13.6596	14.399	6.264945
C	12.4122	13.663	6.24243
C	7.5848	16.5325	6.442095
C	6.3384	15.80325	6.43185
C	9.9966	16.54425	6.578115
C	8.818	15.808	6.580155
C	12.4124	16.524	6.425595
C	11.1676	15.8045	6.424755
C	13.661	15.803	6.2814

C	8.8182	18.76875	6.520455
C	7.5868	17.9505	6.43356
C	11.1772	18.74075	6.694965
C	9.9974	17.94975	6.69297
C	12.4128	17.948	6.60426
C	8.1034	6.92675	9.379425
C	10.4768	6.84825	9.45837
C	9.281	6.11775	9.486255
C	12.9714	6.858	9.43896
C	11.721	6.12525	9.41457
C	6.8944	9.0735	9.38031
C	9.3098	9.022	9.387315
C	8.0854	8.29975	9.41652
C	11.72	8.98275	9.415065
C	10.4756	8.268	9.410115
C	14.2194	9.0045	9.452595
C	12.9708	8.26725	9.415545
C	8.1468	11.12675	9.514635
C	6.9028	10.4175	9.468885
C	10.5446	11.16275	9.552225
C	9.3768	10.41875	9.483045
C	12.9716	11.12525	9.4164
C	11.7252	10.41175	9.463095
C	14.2196	10.408	9.413895
C	6.8976	13.2905	9.468555
C	9.3372	13.2915	9.511275
C	8.1406	12.5555	9.510255
C	11.724	13.2695	9.468675
C	10.5136	12.56075	9.552255
C	14.2188	13.29075	9.464205
C	12.9714	12.55475	9.458295
C	8.144	15.42425	9.46944
C	6.8976	14.695	9.42144
C	10.5558	15.436	9.39339
C	9.3774	14.7	9.446595
C	12.9716	15.41575	9.458565
C	11.7268	14.6965	9.41013
C	14.2202	14.69475	9.47166
C	9.3774	17.66075	9.545085
C	8.146	16.84225	9.513525
C	11.7364	17.6325	9.404235
C	10.5566	16.84175	9.548565
C	12.972	16.83975	9.413295

O	8.6596	5.9335	6.69273
O	11.161	5.977	6.269235
O	8.8388	20.0505	6.556665
O	11.1746	19.999	6.696315
O	9.2188	4.8255	9.731055
O	11.7202	4.869	9.37965
O	9.398	18.94225	9.378405
O	11.7338	18.89075	9.375
H	6.584	7.51925	6.596445
H	13.4064	7.48725	6.499725
H	14.6588	9.6545	6.474135
H	5.4634	12.155	6.59697
H	14.6454	12.03175	6.56805
H	5.3386	13.9465	6.59544
H	14.6576	13.94475	6.40542
H	5.3618	16.3575	6.59688
H	14.6362	16.358	6.54993
H	6.5958	18.48575	6.59712
H	13.403	18.48725	6.603795
H	5.341	9.763	6.430905
H	7.1432	6.411	9.375
H	13.9656	6.379	9.316275
H	15.218	8.54625	9.459435
H	6.0226	11.04675	9.46578
H	15.2046	10.9235	9.43755
H	5.898	12.83825	9.465285
H	15.2168	12.83675	9.456705
H	5.921	15.2495	9.458235
H	15.1954	15.24975	9.4713
H	7.155	17.3775	9.46599
H	13.9622	17.379	9.46317
H	5.9002	8.65475	9.30018

Optimized Cartesian coordinates (Å) of stacking mode SP-2.

C	7.5442	8.03475	6.52122
C	9.9176	7.95625	6.427605
C	8.7218	7.226	6.523875
C	12.4122	7.96625	6.421695
C	11.1618	7.2335	6.427215
C	6.3352	10.1815	6.51369
C	8.7506	10.13	6.511875
C	7.5262	9.408	6.515445

C	11.1608	10.091	6.278535
C	9.9164	9.375	6.42351
C	13.6602	10.11275	6.408645
C	12.4116	9.375	6.339
C	7.5876	12.235	6.4272
C	6.3436	11.52575	6.343275
C	9.9854	12.27075	6.48687
C	8.8176	11.52675	6.51513
C	12.4124	12.2335	6.25005
C	11.166	11.52	6.338265
C	13.6604	11.516	6.28143
C	6.3384	14.39875	6.56925
C	8.778	14.39975	6.511725
C	7.5814	13.66375	6.443415
C	11.1648	14.37775	6.256245
C	9.9544	13.669	6.38094
C	13.6596	14.399	6.40989
C	12.4122	13.663	6.251175
C	7.5848	16.5325	6.430155
C	6.3384	15.80325	6.34416
C	9.9966	16.54425	6.57717
C	8.818	15.808	6.58149
C	12.4124	16.524	6.341025
C	11.1676	15.8045	6.414315
C	13.661	15.803	6.428085
C	8.8182	18.76875	6.51948
C	7.5868	17.9505	6.43251
C	11.1772	18.74075	6.63252
C	9.9974	17.94975	6.693285
C	12.4128	17.948	6.43263
C	8.8056	8.05025	9.37698
C	11.179	7.97175	9.46779
C	9.9832	7.24125	9.46281
C	13.6734	7.9815	9.420075
C	12.4232	7.24875	9.462225
C	7.5966	10.197	9.375
C	10.012	10.1455	9.428625
C	8.7876	9.42325	9.378585
C	12.4222	10.10625	9.46662
C	11.1778	9.3915	9.462885
C	14.9216	10.128	9.41826
C	13.673	9.39075	9.458625
C	8.849	12.25025	9.47004

C	7.605	11.541	9.45036
C	11.2468	12.28625	9.39786
C	10.079	11.54225	9.451185
C	13.6738	12.24875	9.46476
C	12.4274	11.53525	9.46041
C	14.9218	11.5315	9.460635
C	7.5998	14.414	9.51021
C	10.0394	14.415	9.376695
C	8.8428	13.679	9.46461
C	12.426	14.393	9.45597
C	11.2158	13.68425	9.378255
C	14.921	14.41425	9.418485
C	13.6736	13.67825	9.461205
C	8.8462	16.54775	9.448815
C	7.5998	15.8185	9.468045
C	11.258	16.5595	9.397965
C	10.0794	15.8235	9.37668
C	13.6738	16.53925	9.469155
C	12.429	15.82	9.46035
C	14.9224	15.81825	9.45903
C	10.0796	18.78425	9.547335
C	8.8482	17.96575	9.472965
C	12.4386	18.756	9.551295
C	11.2588	17.96525	9.551535
C	13.6742	17.96325	9.45669
O	8.6596	5.9335	6.692115
O	11.161	5.977	6.4284
O	8.8388	20.0505	6.51327
O	11.1746	19.999	6.69618
O	9.921	5.949	9.51597
O	12.4224	5.9925	9.4647
O	10.1002	20.06575	9.497715
O	12.436	20.01425	9.553845
H	6.584	7.51925	6.596115
H	13.4064	7.48725	6.500205
H	14.6588	9.6545	6.515775
H	5.4634	12.155	6.592545
H	14.6454	12.03175	6.567945
H	5.3386	13.9465	6.61713
H	14.6576	13.94475	6.498435
H	5.3618	16.3575	6.56544
H	14.6362	16.358	6.59703
H	6.5958	18.48575	6.59412

H	13.403	18.48725	6.596145
H	5.341	9.763	6.43074
H	7.8454	7.5345	9.42285
H	14.6678	7.5025	9.380355
H	15.9202	9.66975	9.38727
H	6.7246	12.17025	9.291135
H	15.9068	12.047	9.459795
H	6.6	13.96175	9.491355
H	15.919	13.96025	9.38661
H	6.6232	16.373	9.463785
H	15.8976	16.37325	9.462705
H	7.8572	18.501	9.4638
H	14.6644	18.5025	9.29646
H	6.6024	9.77825	9.259965

Supporting References

[1] S. Yoo, B. Domercq. B. Kippelen, *Appl. Phys. Lett.* **2004**, *85*, 5427.

[2] Q. Zhang, B. Kan, F. Liu, G. Long, X. Wan, X. Chen, Y. Zuo, W. Ni, H. Zhang, M. Li, Z. Hu, F. Huang, Y. Cao, Z. Liang, M. Zhang, T. Russell, Y. Chen, *Nat. Photonics* **2015**, 9. 35.

[3] P. E. Shaw, A. Ruseckas, I. D. W. Samuel, *Adv. Mater.* **2008**, *20*, 3516.

[4] P. Bi, S. Zhang, Z. Chen, Y. Xu, Y. Cui, T. Zhang, J. Ren, J. Qin, L. Hong, X. Hao, J. Hou, *Joule* **2021**, *5*, 2408.

[5] H. Lu, K. Chen, R. S. Bobba, J. Shi, M. Li, Y. Wang, J. Xue, P. Xue, X. Zheng, K. E. Thom, I. Wagner, C. Lin, Y. Song, W. Ma, Z. Tang, Q. Meng, Q. Qiao, J. M. Hodgkiss, X. Zhan, *Adv. Mater.* **2022**, *34*, 2205926.

[6] Y. Shi, Y. Chang, K. Lu, Z. Chen, J. Zhang, Y. Yan, D. Qiu, Y. Liu, M. A. Adil, W. Ma, X. Hao, L. Zhu, Z. Wei, *Nat. Communi.* **2022**, 3, 3256.

[7] R. Sun, Y. Wu, X. Yang, Y. Gao, Z. Chen, K. Li, J. Qiao, T. Wang, J. Guo, C. Liu, X. Hao, H. Zhu, J. Min, *Adv. Mater.* **2022**, *34*, 2110147.
